# Allele-resolved monosome and polysome sequencing identifies cis-variants affecting mRNA translation

**DOI:** 10.26508/lsa.202603847

**Published:** 2026-10-07

**Authors:** Laura Alunno, Ilaria Massignani, Meriem Hadjer Hamadou, Fabio Mazza, Daniele Peroni, Romina Belli, Erik Dassi, Alessandro Romanel, Alberto Inga

**Affiliations:** 1 Laboratory of Transcriptional Networks, Department of Cellular, Computational, and Integrative Biology, CIBIO, University of Trento , Trento, Italy; 2 Laboratory of Bioinformatics and Computational Genomics, Department of Cellular, Computational, and Integrative Biology, CIBIO, University of Trento , Trento, Italy; 3 Mass Spectrometry Core Facility, Department of Cellular, Computational, and Integrative Biology, CIBIO, University of Trento , Trento, Italy; 4 Laboratory of RNA Regulatory Networks, Department of Cellular, Computational, and Integrative Biology, CIBIO, University of Trento , Trento, Italy

## Abstract

To prioritize germline genetic variants affecting mRNA fate at the translational level, we used sucrose gradient–based isolation of 80S monosomes and polysomes, followed by RNA sequencing in RPE-1 cells, under mock and nutlin treatment conditions. Differential gene expression analysis confirmed a canonical p53 response. Heterozygous SNPs and SNVs were identified from the RNA-sequencing data, and allelic fractions were calculated for total, monosomal, and polysomal mRNAs. Variants showing reproducible allelic fraction differences across fractions beyond experimental variability were defined as tranSNPs. Among over 7,000 heterozygous variants analyzable in polysomal RNA and over 5,000 in mRNAs associated with monosome-enriched fractions, 1,247 in 1,015 genes displayed a significant imbalance. Reporter assays performed in RPE-1 and HCT116 cells validated allelic or haplotype effects for 17 selected variants in UTRs and coding regions, confirming differences in 15 cases, with evidence of cell line–specific responses. Proteomic analysis supported allelic imbalance for a few selected missense variants. Monosome-enriched fractions improved sensitivity in measuring allelic imbalances without introducing a tranSNP positional bias, suggesting that 80S profiling enhances detection of allele-specific translational regulation in RPE-1 cells.

## Abstract (precis)

Allele-specific profiling of ribosome-associated mRNAs reveals widespread cis-regulatory effects on translation and shows that 80S monosome analysis improves detection sensitivity.

## Graphical Abstract

**Figure:**
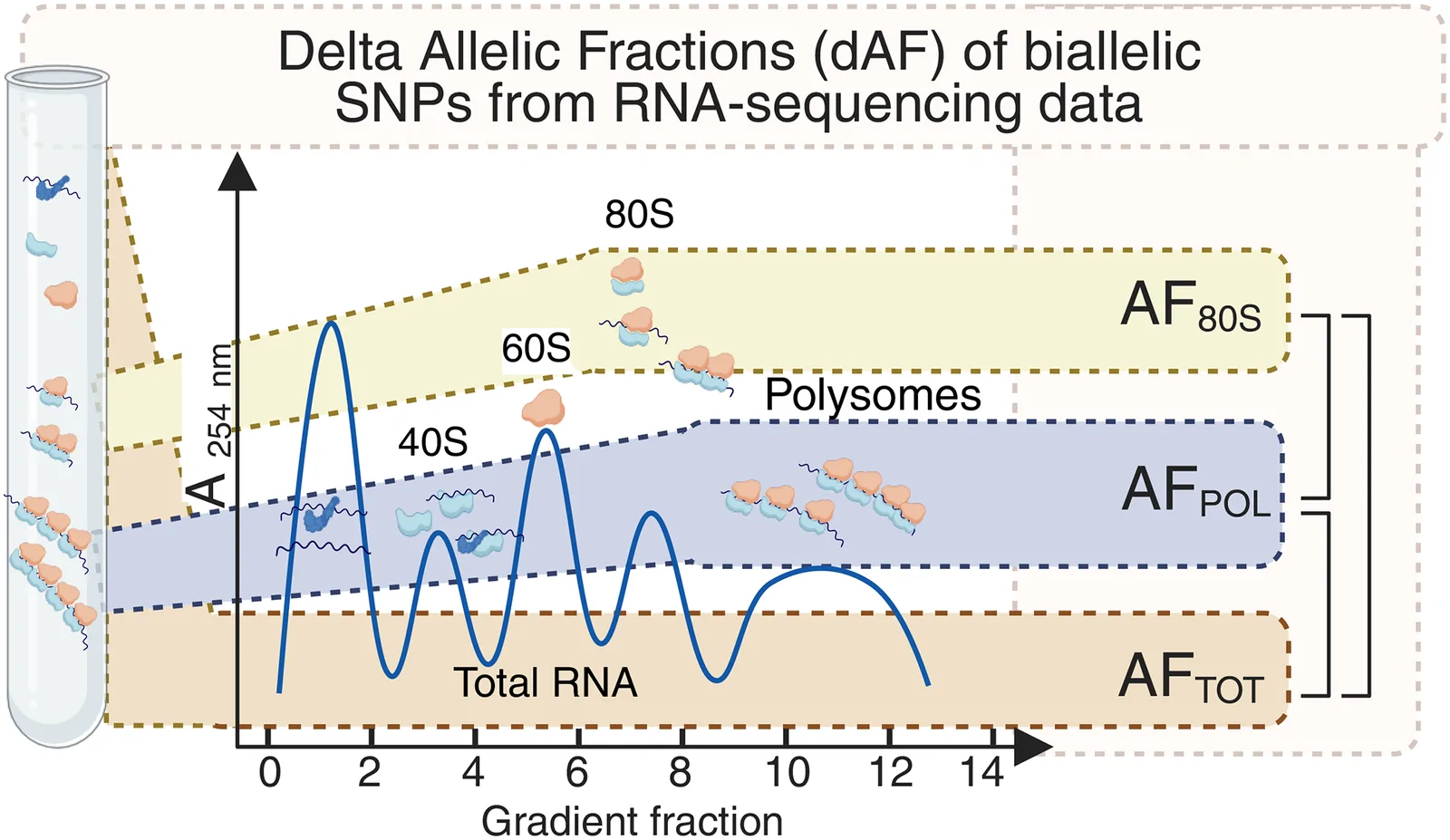

## Introduction

The relevance of the posttranscriptional and translational controls in modulating gene expression has recently been emphasized (Mata et al, 2005; Ruggero, 2013; Zhao et al, 2016; Robichaud et al, 2019; Jia et al, 2020; Mitschka & Mayr, 2022; Orellana et al, 2022; Wu & Bazzini, 2023; Biffo et al, 2024). However, only a limited number of studies have examined genetic variants affecting posttranscriptional events (Robert & Pelletier, 2018; Li & Chen, 2023; Huang et al, 2025; Zheng et al, 2025). Nonetheless, specific examples of functional SNPs affecting mRNA translation in disease-relevant genes, such as *KRAS*, *TP53*, or *FGF20*, have been identified (Chin et al, 2008; Wang et al, 2008; Khan et al, 2013). Furthermore, integrative genome-scale studies comparing RNA expression, translation, and protein output or exploiting massively parallel translation assays have revealed many single-nucleotide variants, including common SNPs, that can affect ribosome loading (Li et al, 2013; Cenik et al, 2015; Sample et al, 2019). However, an established experimental pipeline for systematic prioritization of functional variants that affect mRNA fate is lacking, although tools using, for example, global translation efficiency data and machine learning to reach nucleotide resolution are emerging (Xiong et al, 2025; Zheng et al, 2025).

We recently developed a method for the identification of tranSNPs, where tranSNPs were defined as “a class of functional SNPs affecting the mRNA translation potential” (Valentini et al, 2021). The approach relies on the comparative analysis of the allelic fraction (AF) of the alternative allele across polysome-bound mRNAs (POL) and total mRNAs (TOT) for the SNPs that are heterozygous and transcribed in a given cell model. POL are considered a proxy for the actively translated mRNAs, whereas TOT represent the global transcript population, by containing both actively translated and poorly or untranslated mRNAs. When two SNP alleles are significantly imbalanced in polysomal RNA compared with TOT, one allele is evidently enriched in the polysomes; hence, the SNP is identified as a putative tranSNP, which we would hypothesize affects the translatability of the SNP-containing mRNA. The pipeline was applied to the MCF7 and HCT116 cancer cell lines, and a total of 187 SNPs were identified as putative tranSNPs, corresponding to about 5% of the analyzable SNPs (Valentini et al, 2021; Hamadou et al, 2025). About 25% of tranSNPs were located in the untranslated regions (UTRs), with the remainder in coding regions, and no enrichment for specific pathways was observed among the genes harboring them. Two tranSNPs were selected for an in-depth validation of their functional effect and significance: rs1053639 on DDIT4 3′ UTR (Hamadou et al, 2025) and the rs1554710467 missense variant on EIF4H coding sequence (CDS), the latter one validated also by allele-specific proteomics (Hamadou et al, 2026).

In this study, we further implemented the tranSNP workflow and applied it to the human retinal pigment epithelial RPE-1 cell line to explore the tranSNP phenomenon in noncancer cells. RPE-1 cells were selected because of their established use in research, transfection, and editing amenability, as well as their well-described and stable karyotypes (Volpe et al, 2025). The RPE-1 dataset was generated using high coverage (on average ∼130 million reads) paired-end sequencing of three biological replicates. The high coverage enabled the identification of a large number of putative tranSNPs, even within the same mRNA, and allowed a broader exploration of the tranSNP effect, exploring haplotype structure and mechanisms. Importantly, an additional fraction (80S), corresponding to the mRNAs bound to the 80S monosome, was analyzed and compared with the well-established POL and with a total cytoplasmic mRNA reconstituted by pooling all sucrose gradient fractions.

The translation competence of the 80S is still somewhat debated, although evidence is accumulating regarding stress conditions, organisms, tissue context, and differentiation processes that can modulate the translation potential of the monosomes, extending early studies proposing that protein synthesis occurred on polysomes (Warner et al, 1963; Heyer & Moore, 2016; Biever et al, 2020; Shetty et al, 2023; Blandy et al, 2025; Rahaman et al, 2025). Although our approach does not directly measure translation, the analysis of allelic imbalances of heterozygous variants between polysomes and 80S could help reveal differences between these two fractions in their engagement with specific transcripts marked by allelic differences in UTRs or coding regions.

## Results

### TranSNPs can be identified in noncancer cells

**Figure 1.**
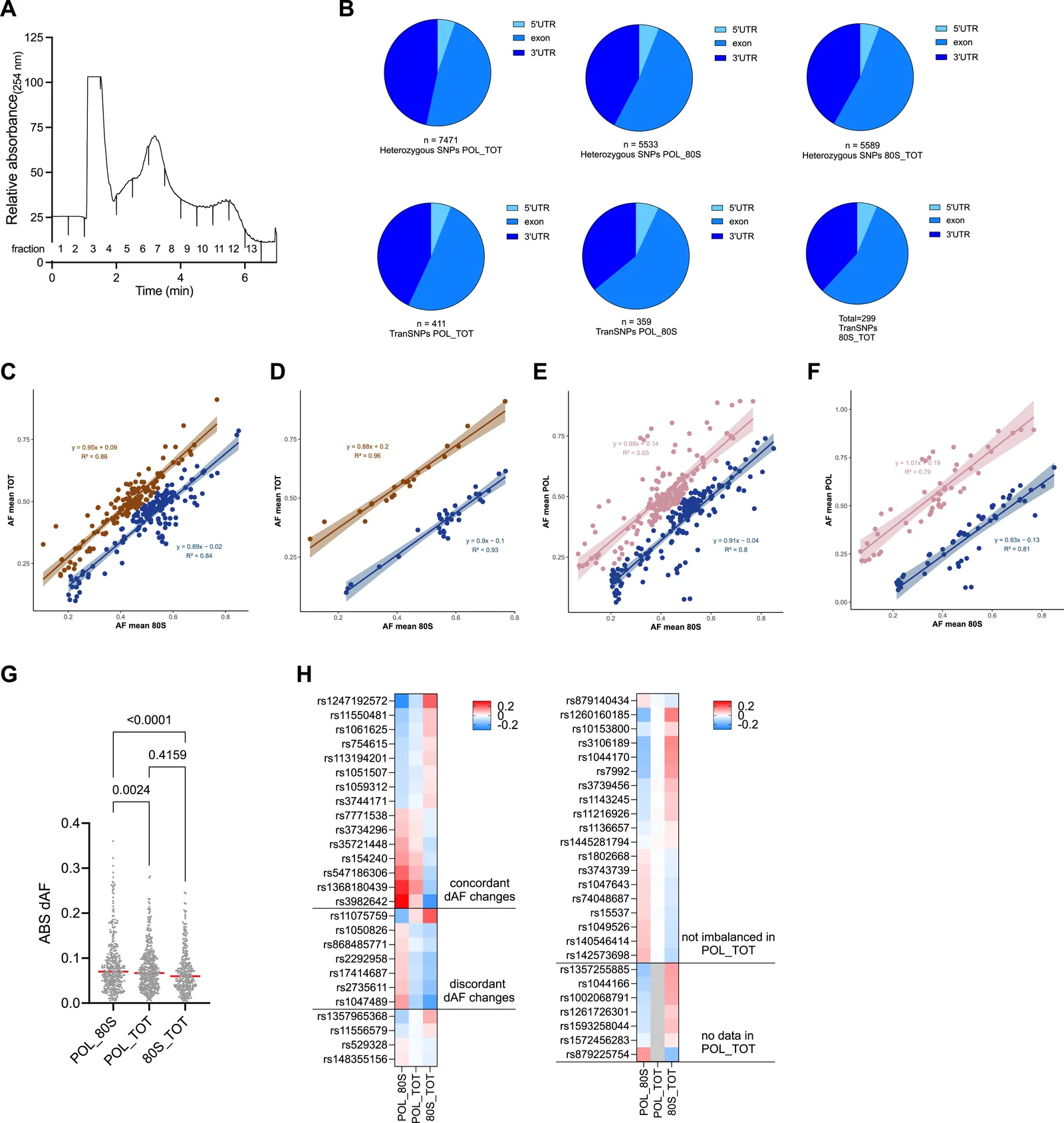
Comparison of POL-80S improves discrimination in tranSNP identification. **(A)** Representative polysomal profile obtained upon sucrose gradient centrifugation of an RPE-1 cell lysate. Fractions 10–13 were pooled together to prepare the polysomal mRNA-sequencing library, fractions 7–9 were pooled to prepare the monosome-enriched library, whereas equal volume samples from each of the 13 fractions were pooled to reconstitute the TOT. **(B)** Pie charts plotting the distribution of either total analyzable SNPs (top) or tranSNPs (bottom) by transcript features (5′ UTR, 3′ UTR or coding/exon). **(C)** Distribution of AF values across 80S and TOT fractions for the tranSNPs selected based on *P* < 0.05. **(D)** Distribution of AF values across 80S and TOT fractions for the tranSNPs selected based on *P* < 0.05 and absolute value >2SD. **(E)** Distribution of AF values across 80S and POL fractions for the tranSNPs selected based on *P* < 0.05. **(F)** Distribution of AF values across 80S and POL fractions for the tranSNPs selected based on *P* < 0.05 and absolute value > 2SD. **(G)** Distribution of absolute AF imbalances (dAF) for tranSNPs across the POL-80S, 80S-TOT, and POL-TOT comparisons. Ordinary one-way ANOVA. **(H)** Heatmap plotting the dAF values for significant tranSNPs both in the POL-80S and in the 80S-TOT fractions. The trends of the corresponding POL-TOT values were also plotted. tranSNPs were divided into four classes based on the concordance of the dAF among POL-80S data or 80S-TOT data with the POL-TOT data.

**Figure S1.**
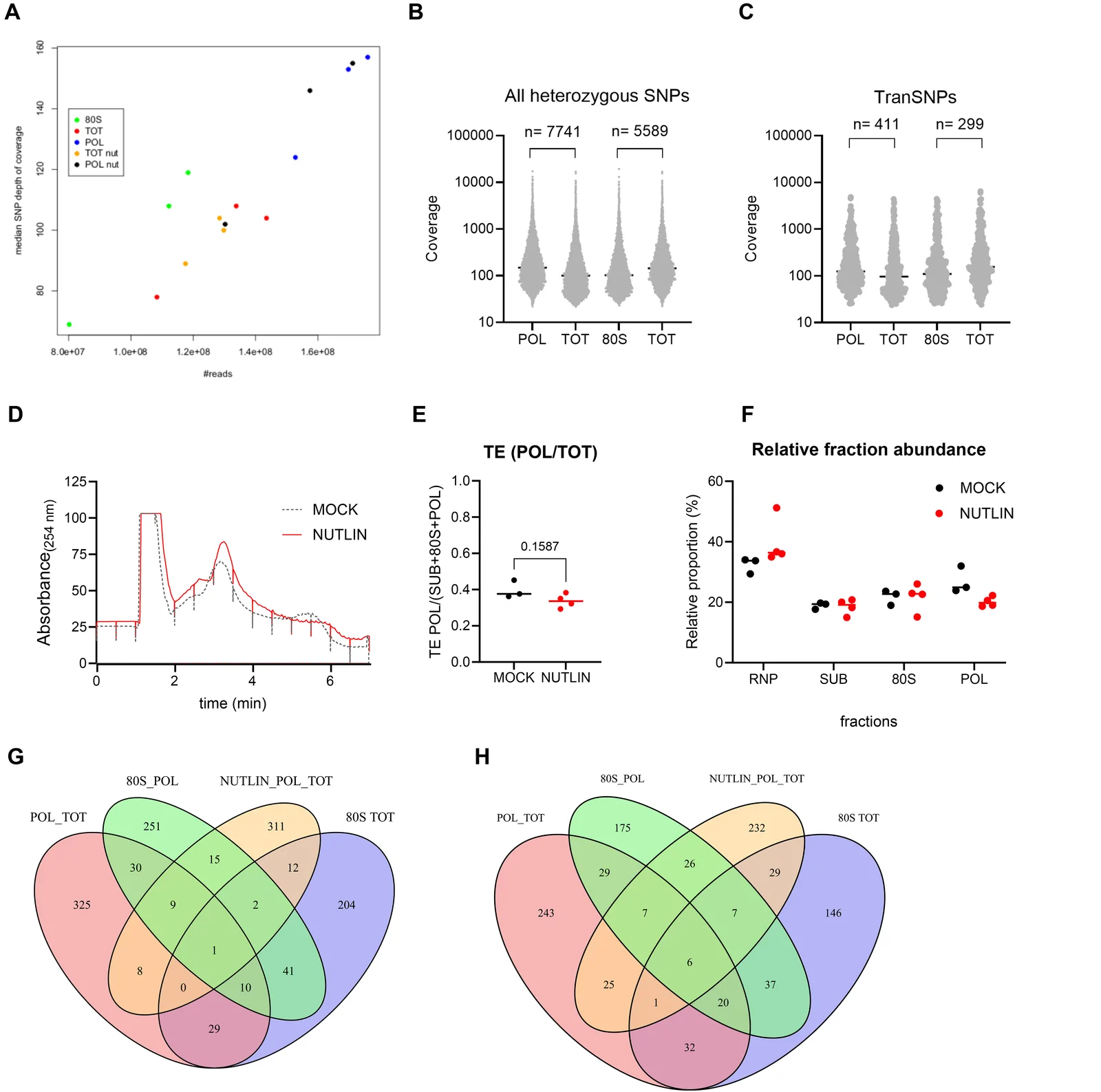
Coverage of the RNA-seq datasets and the global impact of nutlin treatment on features of the polysomal profile and global translation (related to Fig 1). **(A)** Plot showing the correlation between the number of reads and the median coverage at heterozygous SNP sites for the different fractions and the individual biological replicates. A positive correlation is seen, as expected. An apparent higher SNP coverage is visible for the 80S datasets (green dots). **(B, C)** Coverage for all analyzable heterozygous SNPs and for the identified tranSNPs, resulting from the polysomal-versus-total RNA comparison (POL-TOT) or the monosomal/80S-versus-total RNA comparison (80S-TOT). The number of SNPs is shown. The black bars highlight the median of the distribution. **(D)** Representative polysomal profile obtained with nutlin-treated (10 M for 16 h) RPE-1 cells. For comparison, the profile obtained in the mock condition (see Fig 1) is overlaid as a dotted gray line. The higher proportion of the 80S peak is visible. **(E)** Estimated global TE from the quantification of the polysomal profile areas, expressed as a ratio between polysomal and total RNA fractions. The individual values and the mean for three biological replicates of each condition are shown. The trend for a reduction in TE in nutlin-treated cells was not significant (*t* test). **(F)** Relative proportion of the indicated fractions of RPE-1 polysomal profiles, related to panel (E). **(G, H)** Venn diagrams showing the overlap between tranSNPs filtered on nominal *P*-values (*P* < 0.05; see Table S1) (G) and tranSNP-harboring genes (H) between the indicated datasets. TE, translation efficiency.

We wondered whether the allelic imbalance that led to the identification of tranSNPs was exclusive to cancer cells because of their broadly rewired cellular deregulation, or whether it also occurred in nontransformed cells. Thus, we generated a new catalog of tranSNPs in the hTERT-immortalized retinal pigment epithelial cells (RPE-1) through polysomal profiling (Fig 1A) and RNA sequencing (RNA-seq), characterized by high coverage (average 134 million reads, Table S1 and Fig S1A) and three biological replicates for each fraction.

Table S1. Lists of analyzable SNPs and identified TranSNPs.

We identified heterozygous SNPs from RNA-seq data by applying a minimum coverage threshold of 20 reads per replicate and condition. The coverage was comparable among fractions (Fig S1B and C). In addition, we filtered variants based on AF ranging between 20 and 80% (Table S1). This threshold has been validated in previous studies (Prandi et al, 2014; Romanel et al, 2015; Valentini et al, 2021; Orlando et al, 2022) and successfully applied across several of our datasets, allowing robust identification of heterozygous SNPs while accommodating moderate allelic imbalance and reference-mapping bias. Then, for analyzable heterozygous SNPs, we calculated the allelic imbalance across POL and total cytoplasmic mRNAs (delta allelic fraction, dAF), that is to say, the difference in allelic frequencies between these two mRNA pools. We used nominal *P*-values derived from paired t tests across biological replicates to prioritize and catalog putative RPE-1 tranSNPs. For these dAF calculations, cases in which AF was within the 20–80% range in only one fraction were included, to avoid missing extreme imbalances. To improve the tranSNP identification pipeline, we added a further fraction to this dataset, represented by 80S monosome-bound mRNAs, and compared it with TOT and POL. We identified 411 tranSNPs in RPE-1 by comparing polysome-bound and total mRNAs (POL-TOT), 359 by comparing polysome-bound and 80S-bound mRNAs (POL-80S), and 299 by comparing the 80S-bound and total mRNAs (80S-TOT), indicating that tranSNPs can be identified in noncancer settings as well (Table S1). We also applied an additional filter of dAF< −2SD or dAF > 2SD to the identified tranSNPs, based on the variance in AF measurements across all analyzable heterozygous SNPs for each condition or treatment, to reduce false positives and prioritize variants with larger allelic imbalance. This approach complements nominal *P-*value–based prioritization by incorporating orthogonal features, including effect size and consistency across replicates, thereby increasing stringency. With this approach, we restricted the tranSNP lists to 73 when comparing POL-TOT, 107 when comparing POL-80S, and 56 when comparing the 80S-TOT (Table S1 and Fig 1E and F).

**Figure S2.**
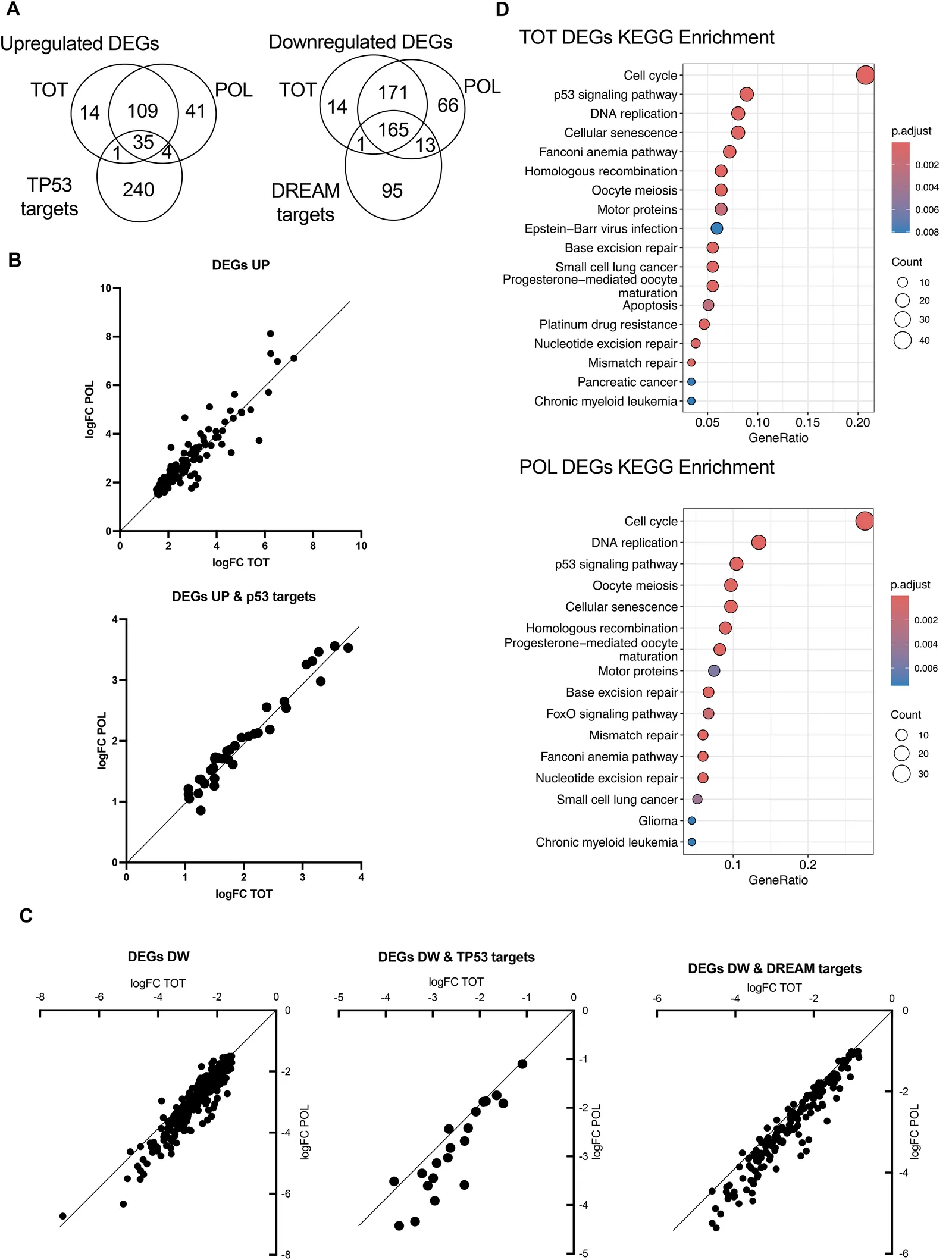
Nutlin treatment induces p53 and cell cycle arrest pathway responses in RPE-1 cells (related to Fig 1). **(A)** Venn diagrams presenting the number of DEGs in response to nutlin treatment in RPE-1 cells and comparing the results obtained with TOT and polysome-associated mRNAs. Left diagram: up-regulated DEGs. Genes were filtered for log_2_ fold change above 1.5 and FDR < 0.05. Most of the DEGs were common to both RNA fractions. The overlap with a curated, extensive list of TP53 target genes derived from the Fisher’s lab is shown. Right diagram: down-regulated DEGs. Genes were filtered for log_2_ fold change below −1.5 and FDR < 0.05. Most of the DEGs were common to both RNA fractions, and there was a substantial overlap with a curated list of DREAM target genes, curated by the Engeland laboratory, that are indirectly repressed by p53. **(B)** Comparison of the fold changes in total (x-axis) and polysomal (y-axis) RNA fractions for up-regulated DEGs. Most of the nutlin-induced gene expression changes are comparable in the transcriptome and the translatome, particularly for direct TP53 target genes (bottom panel). **(C)** Comparison of the fold changes in total (x-axis) and polysomal (y-axis) RNA fractions for down-regulated DEGs. A stronger relative repression in polysomal RNA is apparent for the entire DEG list and for established TP53 (center panel) or DREAM targets (right), possibly consistent with a general inhibition of translation caused by nutlin treatment (Attardi laboratory). **(D)** KEGG semantic enrichment for the DEGs confirming the broad similarity among transcriptome and translatome data and the strong enrichment for cell cycle arrest and p53 activation signatures. FDR, false discovery rate; DEGs, differentially expressed genes.

In addition to the mock condition, an experiment with nutlin-treated cells (10 μM for 16 h) was performed. Nutlin, through MDM2 inhibition and wild-type p53 activation, is known to elicit broad changes not only at the transcriptional but also at the posttranscriptional and translational regulation levels (Rizzotto et al, 2020; Tiu et al, 2021). Consistently, the treatment increased the already abundant 80S peak in the RPE-1 sucrose gradient polysomal profile (Fig S1D) and slightly decreased the relative abundance of polysomes (Fig S1E and F). Differential gene expression analyses confirmed that the treatment activated the p53 and cell cycle arrest pathways in both TOT and polysomal RNAs, leading to a generalized repression of DREAM complex targets (Fig S2A–D). We identified 358 tranSNPs in RPE-1 by comparing POL-TOT in nutlin-treated cells (Table S1).

In total, 1,247 unique tranSNPs were found. Their distribution by transcript features was equivalent to that of the entire group of analyzable heterozygous SNPs, with similar proportions of instances in coding and 3′ UTRs and a small proportion in 5′ UTRs (Fig 1B). 135 SNPs were identified in two comparisons, 21 in three different comparisons, and one (rs1051507) in all four comparisons (Fig S1G). The 1,247 unique tranSNPs are associated with 1,015 unique genes. The overlap between lists is higher at the gene level, with 26 tranSNP-containing genes common to all mock comparisons, six of them also common to the nutlin-treated polysome-versus-total comparison (Fig S1H).

### The use of 80S-bound mRNAs improves resolution in the identification of tranSNPs

**Figure S3.**
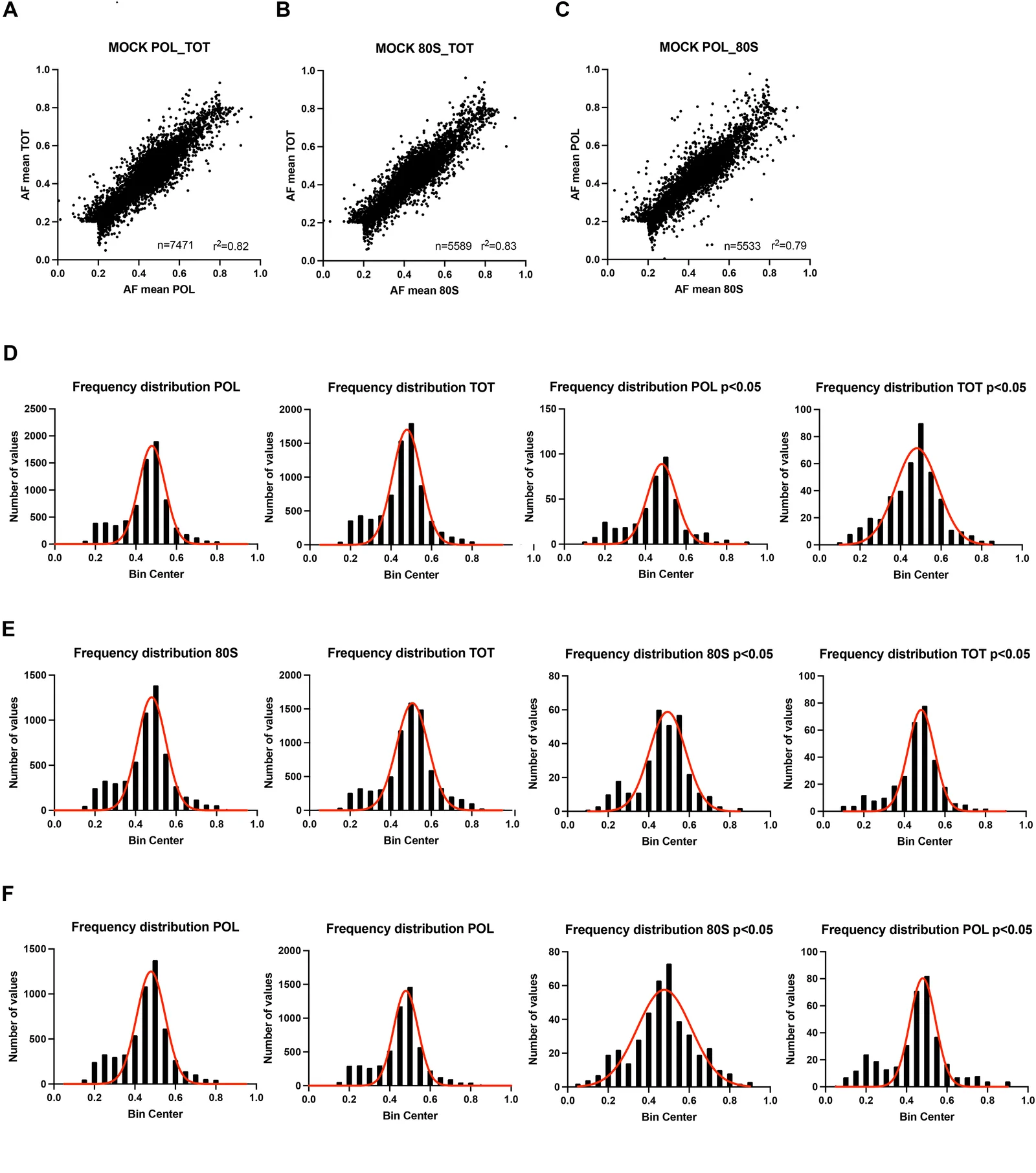
AF distribution across 80S, POL, and TOT comparisons (related to Fig 1 and Table S1). **(A)** Distribution of AF values across the 7,471 analyzable SNPs when comparing polysomal mRNAs and TOT displayed as a scatter plot for correlative analyses. **(B)** Distribution of AF values across the 5,589 analyzable SNPs when comparing 80S-TOT displayed as a scatter plot for correlative analyses. **(C)** Distribution of AF values across the 5,533 analyzable SNPs when comparing 80S-POL displayed as a scatter plot for correlative analyses. **(D)** Distribution of AF values across analyzable SNPs and tranSNPs (*P* < 0.05) for polysomal mRNAs and TOT. **(E)** Distribution of AF values across analyzable SNPs and tranSNPs (*P* < 0.05) for 80S and TOT. **(F)** Distribution of AF values across analyzable SNPs and tranSNPs (*P* < 0.05) for 80S-POL.

**Figure S4.**
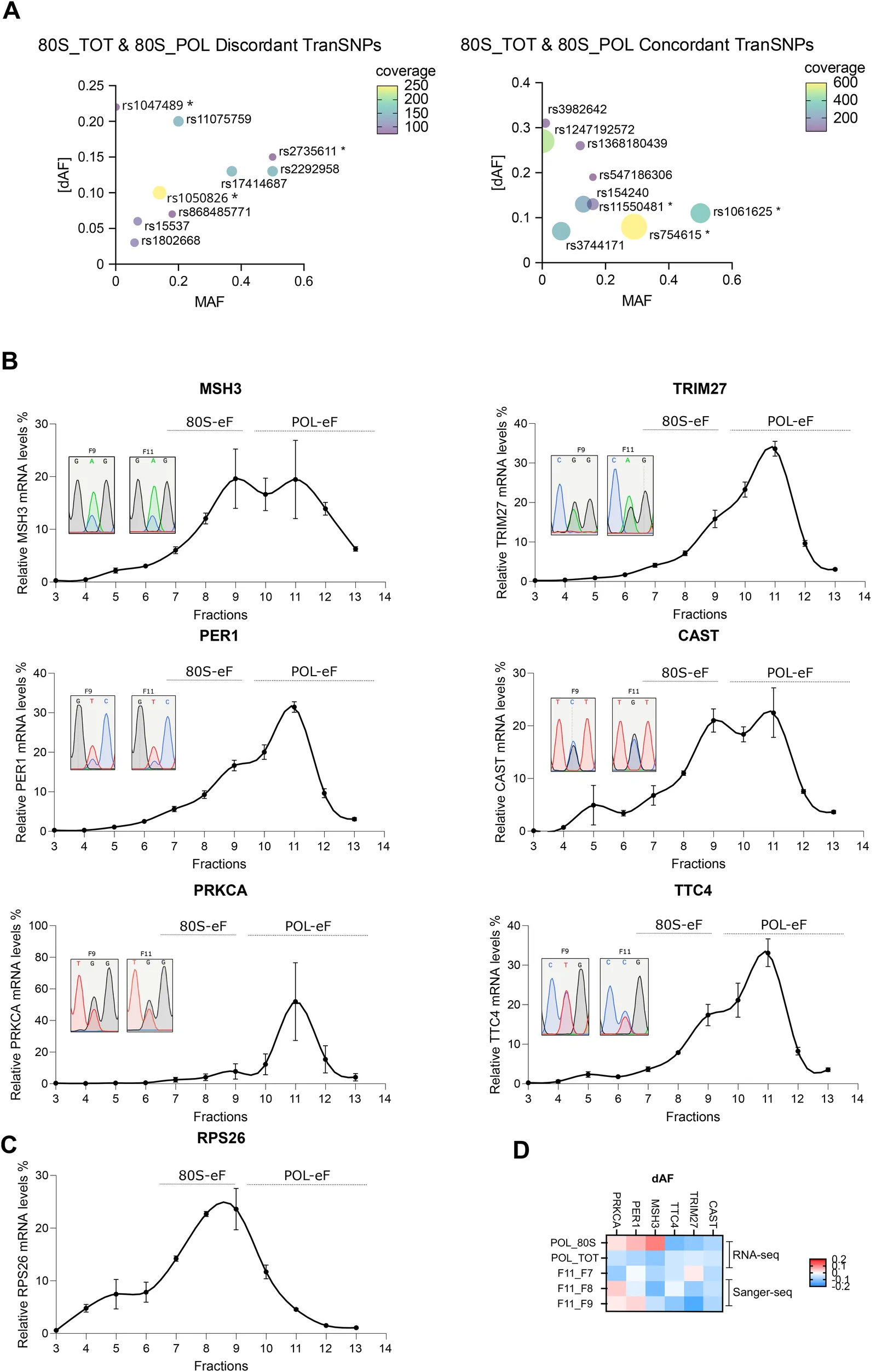
Relative polysomal associations for selected tranSNP genes and orthogonal validation of delta allelic imbalances (related to Fig 1). **(A)** Comparison of the magnitude of delta allelic imbalance, minor allele frequency, and sequence coverage (size and colors of the circles) for the group of identified tranSNPs with discordant allelic imbalance in the comparison indicated in the image titles, and a comparable sample of the concordant group. Concordance and discordance data are presented in Fig 1H. **(B)** Each plot shows the mean relative proportion and the standard deviations of three qRT-PCR technical replicates for the indicated transcripts in each fraction of the sucrose gradient polysomal profile of mock RPE-1 cells. The left and right columns show results for three dAF-discordant and three dAF-concordant targets, respectively. Fractions F7, F8, and F9 are marked as monosome-enriched fractions (80S-eF), whereas fractions F10–F13 are marked as polysome-enriched fractions (POL-eF). These groupings correspond to the pools made for the RNA-seq libraries—a polysomal profile based on the A_254nm_ absorbance data is visible in Fig 1A. Snapshots of the Sanger electropherograms centered at each heterozygous tranSNP, for amplicons obtained from F9 and F11 cDNAs, are overlaid to each mRNA profile. **(C)** Profile of the *RPS26 *TOP mRNA is shown as representative of a transcript exhibiting low translation efficiency. **(D)** Comparison of the dAF values obtained for the six targets in the RNA-seq datasets and calculated from Sanger sequencing electropherograms, using data from fraction 11, corresponding to polysomes, compared with either F7, F8, or F9.

We used the RPE-1 dataset to evaluate the allelic imbalance (dAF) of tranSNPs, focusing for the first time also on the 80S monosome data. By comparing the correlation between AF in the 80S-TOT and POL-80S datasets, expressed as R^2^ scores, we found that the POL-80S comparison better discriminated changes in AF imbalances (Figs 1C–F, and S3A–F). Notably, all 52 tranSNPs shared between the 80S-TOT and POL-80S comparisons displayed complementary delta allelic imbalance (Fig 1H). This systematic observation led us to think that the 80S fraction and the polysomal fraction represent extreme conditions for dAF measurements, whereas POL-TOT dAF values (even when statistical significance was not reached) fell in the middle. Interestingly, we found that the percentage of tranSNPs relative to analyzable SNPs was higher in the 80S-POL (about 6.5% based on nominal *P*-values, or 1.9% after applying variance-based filtering) than in 80S-TOT (about 5.4% or 1%) or POL-TOT (5.5% or 1%) comparisons. These data further supported the hypothesis that 80S monosomes are a better proxy for unmasking allelic differences in mRNA translation than total cytoplasmic mRNAs, at least in RPE-1 cells, providing higher resolution in the identification of tranSNPs through our pipeline. Similarly, a comprehensive analysis of AF imbalances across the 80S-POL, POL-TOT, and 80S-TOT comparisons confirmed significantly higher magnitude of imbalance in the 80S-POL comparison (Fig 1G). Only 22 of the 52 tranSNPs shared across comparisons that included the 80S dataset showed imbalance in the POL-TOT comparison. Of these, 15 exhibited smaller but concordant dAF changes among POL-80S and POL-TOT datasets, whereas seven were discordant, suggesting that a component of the sample that is unique to the reconstituted total cytoplasmic fraction is driving the allelic imbalance toward both comparisons with 80S and polysomes (Fig 1H). We selected three transcripts from the concordant group (*TRIM27*, *CAST*, and *TTC4*) and three from the discordant group (*PER1*, *PRKCA*, and *MSH3*), considering coverage, magnitude of dAF, and minor allele frequencies of the associated tranSNP, and examined transcript distributions along the polysomal profile by qRT-PCR (Fig S4A). Although the results revealed differences in the relative abundance of those transcripts across subpolysomal (F3-F6), monosome-enriched (F7-F9), and polysomal (F10-F13) fractions, these could not be grouped by the dAF concordance or discordance parameter (Fig S4B). RPS26 was included as a top mRNA control (Fig S4C). We also amplified transcript regions encompassing the tranSNPs (for F7, F8, F9, and F11) and used Sanger sequencing as an orthogonal method to quantify allelic imbalance, with F11 as the polysomal fraction. We found that dAF values were highly consistent with the RNA-seq results, particularly for the POL-80S comparison relative to the F11-F8 and F11-F9 comparisons (Fig S4D).

**Figure S5.**
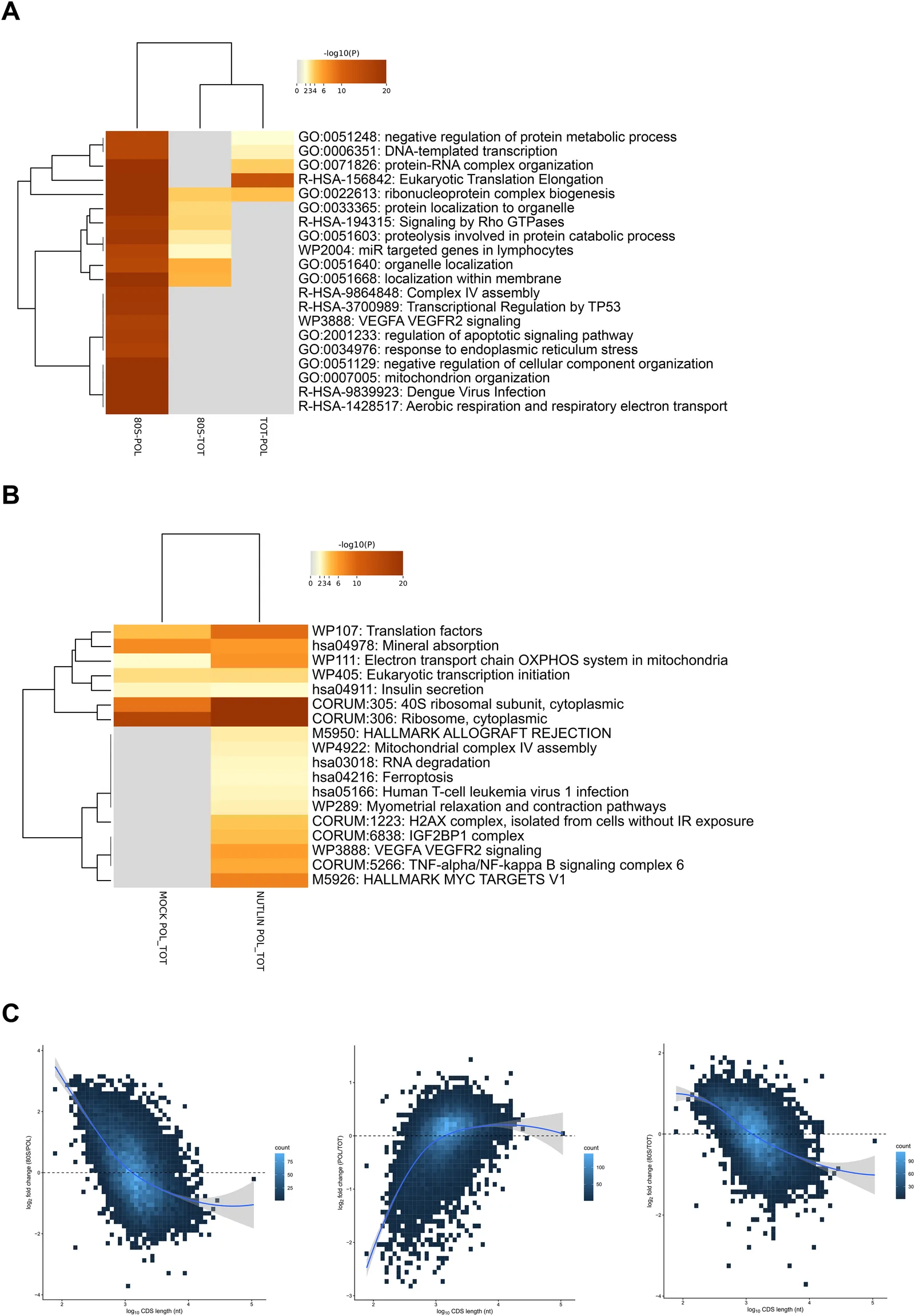
Semantic enrichment, overlap, and correlations with size of CDS among differentially expressed genes across RNA fractions. **(A)** Metascape heatmap view of the Gene Ontology–term enrichment for genes that were differentially expressed in the comparison between monosomal, polysomal, and total RNA fractions. See Methods for details. **(B)** Metascape heatmap view of the Gene Ontology–term enrichment for genes that were differentially expressed between polysomal and total RNA fractions either in the mock or in the nutlin treatment conditions. **(C)** Correlation between the length of the CDS (x-axis, expressed in log10) and fold changes respectively for 80S-POL, POL-TOT, and 80S-TOT comparisons from left to right. A relatively strong negative correlation of CDS length of canonical transcripts with the log_2_ fold change for the 80S-versus-polysome comparison (Spearman’s ρ = −0.53, *P* < 2.2 × 10^−16^).

Although limited by potential heterogeneities in the overall composition and complexity of the fractions, we explored differential gene expression changes among fractions (Fig S5A). Considering the enrichment of Gene Ontology terms, the comparison between 80S and polysomes revealed higher divergence, compared with 80S-TOT or POL-TOT comparisons. However, this apparent divergence should be interpreted with caution, as the larger number of differentially expressed genes detected in the 80S-POL comparison may partly reflect greater statistical power resulting from lower variability among biological replicates.

The comparison between TOT and polysomal mRNAs, both in the mock and in the nutlin-treated conditions, showed enrichment for terms related to cytoplasmic translation and structural constituents of the ribosome in the 80S fraction compared with polysomes (Fig S5B). These data are consistent with the observation that TOP mRNAs have lower translation efficiency or can be translated by monosomes more than polysomes (Thoreen et al, 2012; Jia et al, 2021; Schneider et al, 2022). We also explored a potential relation between the length of the CDS and relative enrichment in 80S or polysomal fractions, as reported previously in yeast (Heyer & Moore, 2016), and found a relatively strong negative correlation of CDS length of canonical transcripts with the log_2_ fold change for the 80S-versus-polysome comparison (Spearmanʼs ρ = −0.53, *P* < 2.2 × 10^−16^) (Fig S5C).

### Haplotype analysis supports the identification of instances of allelic imbalance in monosome sequencing

**Figure 2.**
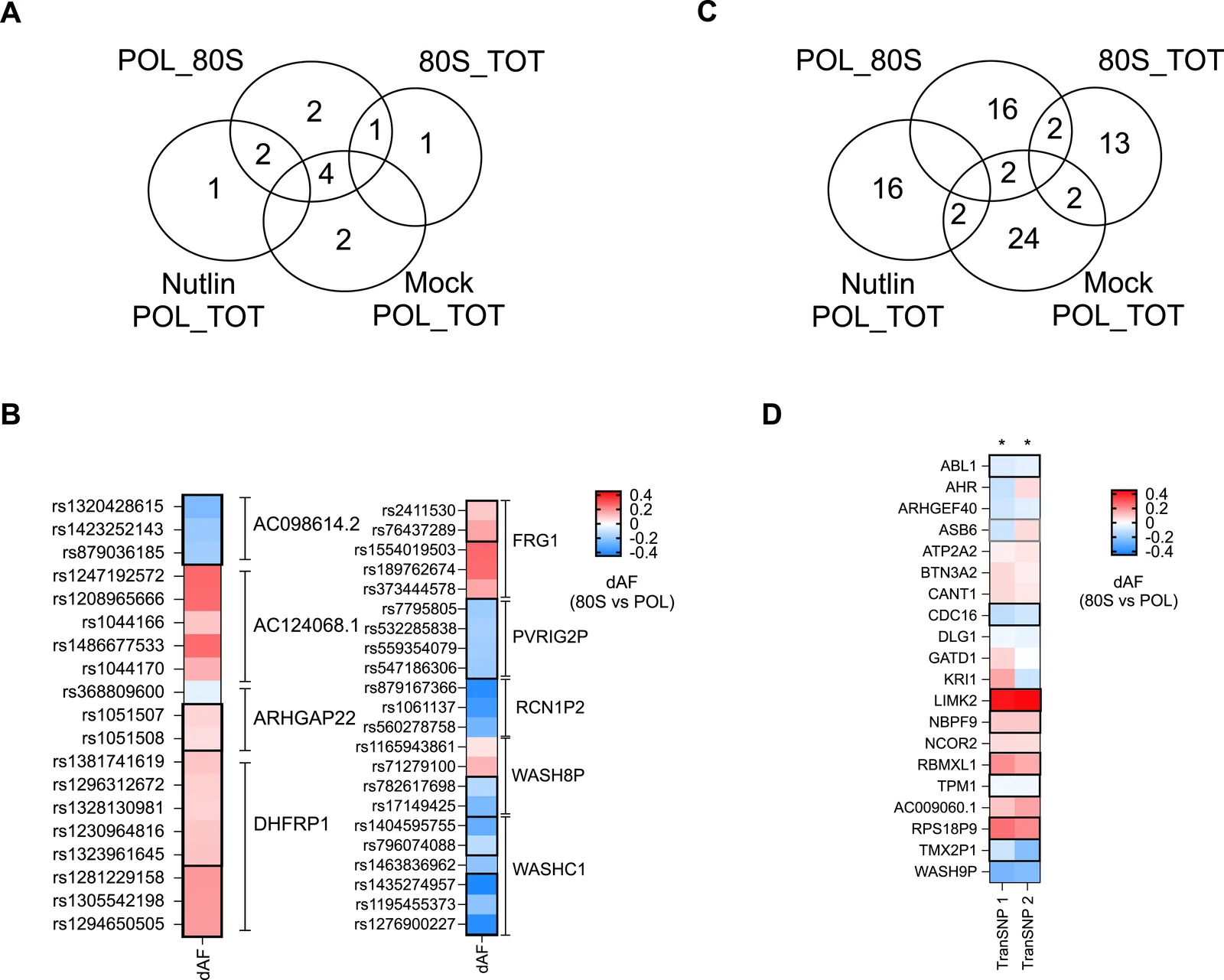
TranSNP haplotype analysis reveals consistent changes in AF. **(A)** Venn diagram presenting the number of tranSNP haplotypes comprising three or more *P* <0.05 SNPs. **(B)** Heatmap view of the dAF values for the nine haplotypes identified as imbalanced in the comparison between 80S monosome and polysome sequencing. Six of those genes are either noncoding RNA or processed pseudogenes. Haplotypes for which phasing data could be retrieved from RNA-seq data are highlighted by thicker boxes; two haplotype blocks were found for both *DHFRP* and *WASHC1* that could not be joined. **(C)** Same as in (A) but focused on tranSNP haplotypes comprising two SNPs. **(D)** Heatmap view of the dAF values for the 16 coding and four noncoding or pseudogene haplotypes identified as imbalanced in the comparison between 80S monosome and polysome sequencing. Haplotypes for which phasing data could be retrieved from RNA-seq data are highlighted by thicker black (in cis) or gray (in trans) boxes. rsID codes are provided in Table S2. * = *P* < 0.05 dAF.

The RNA-seq depth of our experiments allowed us to study over 5 K heterozygous SNPs in the 80S monosome sequencing. Hence, we explored the consistency of allelic imbalance quantifications, exploiting haplotype phase reconstruction. For this analysis, we used the lists of putative tranSNPs (411 for the POL versus TOT, 359 for 80S versus POL, 299 for 80S versus TOT, and 358 for nutlin POL versus TOT). We retrieved haplotypes consisting of at least three SNPs. Consistent with the proposal that monosomes and polysomes display the greatest differences in translation, their comparison uncovered the highest number of tranSNP haplotypes (9, comprising 41 SNPs; Fig 2A). Comparing dAF values within those haplotypes and considering their phase inferred from our RNA-seq data (Table S2), we observed high consistency in the directions of the imbalance data (Fig 2B). Information was retrieved for nine haplotypes across seven genes, totaling 26 SNPs, all of which were cis-haplotypes (i.e., one with all reference alleles, the other with all alternative alleles). We then extended the analysis to genes with two identified tranSNPs (Fig 2C; SNPs marked by an asterisk). A total of 77 haplotypes were identified, of which 20 genes were identified in the 80S-versus-POL comparison (corresponding to about 3.5% of analyzable haplotypes comprising two SNPs) (Fig 2D). Phasing data were inferred from our RNA-seq data for nine pairs, of which eight were in cis and one, *ASB6*, in trans, again showing consistency with the dAF measurements (Fig 2D). Although the actual translation potential of some of these transcripts, which correspond to processed pseudogenes or are annotated as long noncoding RNAs, would need to be formally demonstrated in further studies, the consistency of dAF calculations was our primary focus for validating the tranSNP pipeline.

Table S2. Haplotype phasing data.

We concluded that haplotype analysis can support the identification of instances of allelic imbalance, thereby extending the catalog of functional tranSNPs.

### UTR RPE-1 tranSNPs were validated by luciferase reporter assays

We performed luciferase reporter assays across 5′ and 3′ UTR tranSNP candidates to validate allele-specific translation efficiencies. We shortlisted candidates based on the magnitude of delta allelic imbalance, frequency of the alleles in the population, relevance of the host gene, and concordance of the imbalance with other SNPs in cis in the same mRNA, as inferred from haplotype analysis.

For the 14 chosen candidates, we cloned 109-nucleotide-long fragments of 5′ or 3′ UTRs containing either the reference or the alternative allele upstream or downstream of a Firefly luciferase reporter plasmid. We performed dual-luciferase assays, using the Renilla luciferase as a normalizer of the Firefly luciferase signal first, and then Firefly mRNA levels to further correct the Firefly/Renilla ratios and focus on the posttranscriptional effect of the allelic variants.

**Figure 3.**
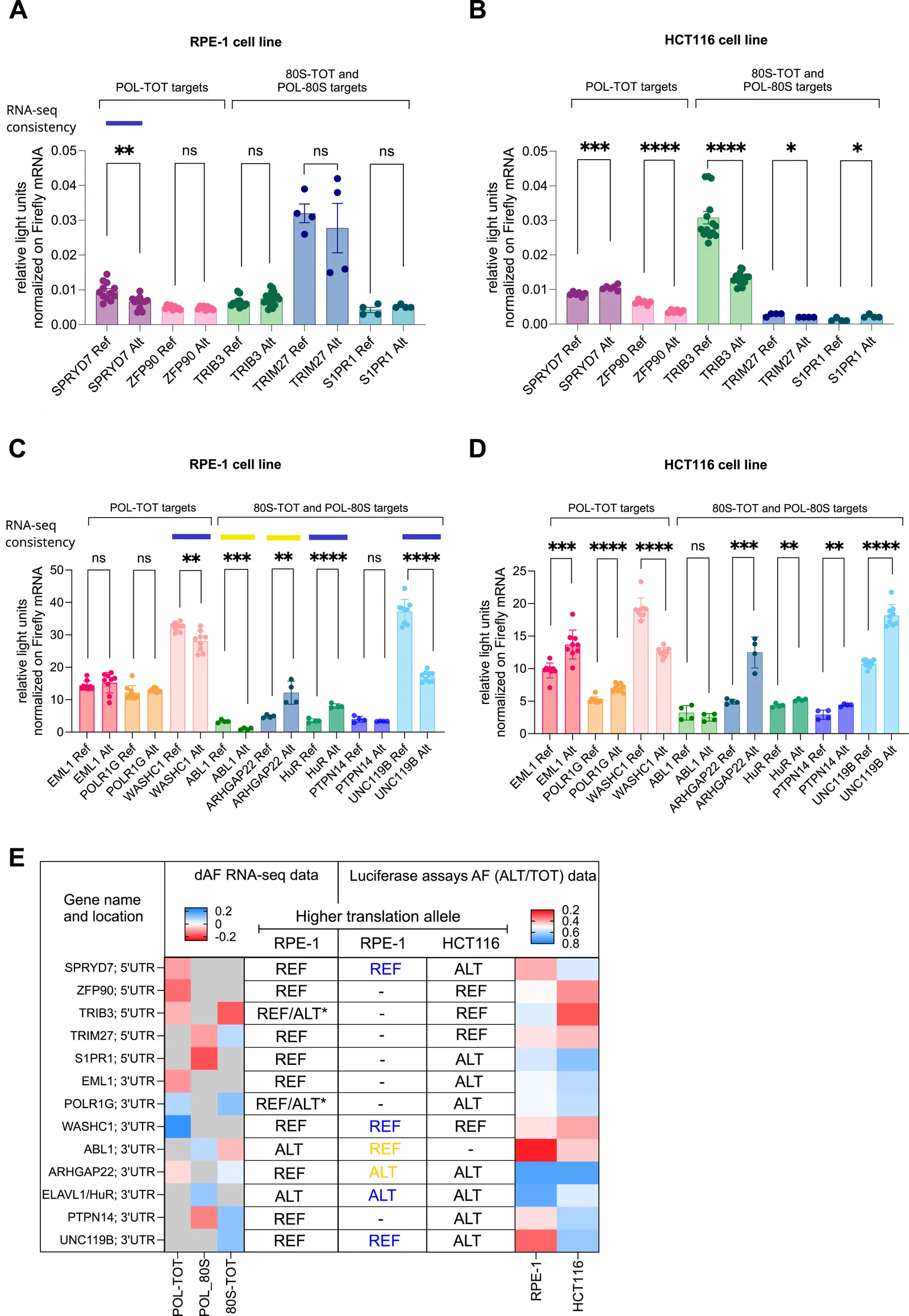
Allele-specific translation efficiencies for UTR RPE-1 tranSNPs were validated by luciferase reporter assays across RPE-1 cells and HCT116 cells. **(A)** Luciferase reporter assay of 5′ UTR constructs encompassing candidate tranSNPs in RPE-1 cells. ns, not significant, **P <0.01, unpaired *t* test. **(B)** Luciferase reporter assay of 5′ UTR constructs encompassing candidate tranSNPs in HCT116 cells. *P <0.05, ***P <0.001, ****P <0.0001, unpaired *t* test. **(C)** Luciferase reporter assay of 3′ UTR constructs encompassing candidate tranSNPs in RPE-1 cells. **P <0.01, ***P <0.001, ****P <0.0001, unpaired *t* test. For the RPE-1 data in panels (A) and (C), the consistency of AF imbalance measured via RNA-seq in RPE-1 and luciferase reporter assay is indicated by color-coded segments. Blue bars indicate consistency, whereas yellow bars indicate an opposite direction of the allelic imbalance. **(D)** Luciferase reporter assay of 3′ UTR constructs encompassing candidate tranSNPs in HCT116 cells. **P <0.01, ***P <0.001, ****P <0.0001, unpaired *t* test. **(E)** Consistency between RNA-seq and luciferase reporter assay data. For the indicated genes, the dAF measured from the RNA-seq data are shown as a heatmap. dAF data were used to infer the allele with higher relative translation efficiency, considering that AF is calculated as ALT/TOT. There are two discordant cases (labeled REF/ALT*) for which the data do not allow to infer the allele exhibiting higher translation potential. The results of the luciferase reporter assays shown in panels (A, B, C, D) are also summarized reporting for each cell line, the tranSNP alleles exhibiting higher activity. For RPE-1, the blue font marks the consistency between dAF and luciferase data (a dash indicates that the difference between the two alleles was not statistically significant). The heatmap on the right plots AF values (ALT/TOT) derived from luciferase activity data for all reporters.

Besides RPE-1 cells, in which this set of tranSNPs was identified, we extended the validation to HCT116 cells, as cancer cells are known to have rewired translation machinery and exhibit different transfection efficiencies (Fernández-Calero et al, 2020). Fig 3A and B shows that most of the 5′ UTR candidates did not exhibit significantly different translation efficiencies in RPE-1 cells, whereas significant differences were observed in HCT116 cells using this assay. Nevertheless, the tranSNP in *SPRYD7* for which we validated allele-specific translation efficiency exhibited consistency in the direction of the allelic imbalance measured by RNA-seq in RPE-1 cells or the luciferase reporter assay in the same cell line (Fig 3A). Notably, the direction of the imbalance was opposite in HCT116 for this candidate (Fig 3B). Instead, we observed a greater extent of significant allele-specific differences among the 3′ UTR candidates in both HCT116 and RPE-1 cells (Fig 3C and D). We retrieved consistent allelic imbalances when comparing RNA-seq and luciferase reporter assay data for three out of five tranSNP host genes in RPE-1 cells (Fig 3C). As cell line differences were expected, we interrogated and detected one more case in which the direction of allelic imbalance was opposite among RPE-1 and HCT116 cells (Fig 3D) possibly because of differences in the availability, levels, or interaction with trans-factors, such as RNA-binding proteins (RBPs), miRNAs, or tRNAs.

We did not find a correlation between the magnitude of allelic imbalance from RNA-seq data and the magnitude of allele-specific translation efficiency from luciferase data (Fig 3E). However, as mentioned earlier, in the reporter assays, the SNP alleles were placed outside their context, and, especially in the 3′ UTR cases, only a small portion of the untranslated sequence from each target was cloned. Although cell line–specific differences suggest a role for trans-factors, tranSNPs could impact RNA structure or create/disrupt uORFs, features that may not be fully recapitulated in an ectopic context. The extensive difference in normalized RLU between the plasmids used for 5′ UTR and 3′ UTR reporter assay is possibly due to distinct transcription and translation features of the differently optimized luciferase CDS present in pGL3 and pGL4 vectors.

### Coding RPE- 1 tranSNPs influence translation in a dual-fluorescence reporter assay

**Figure 4.**
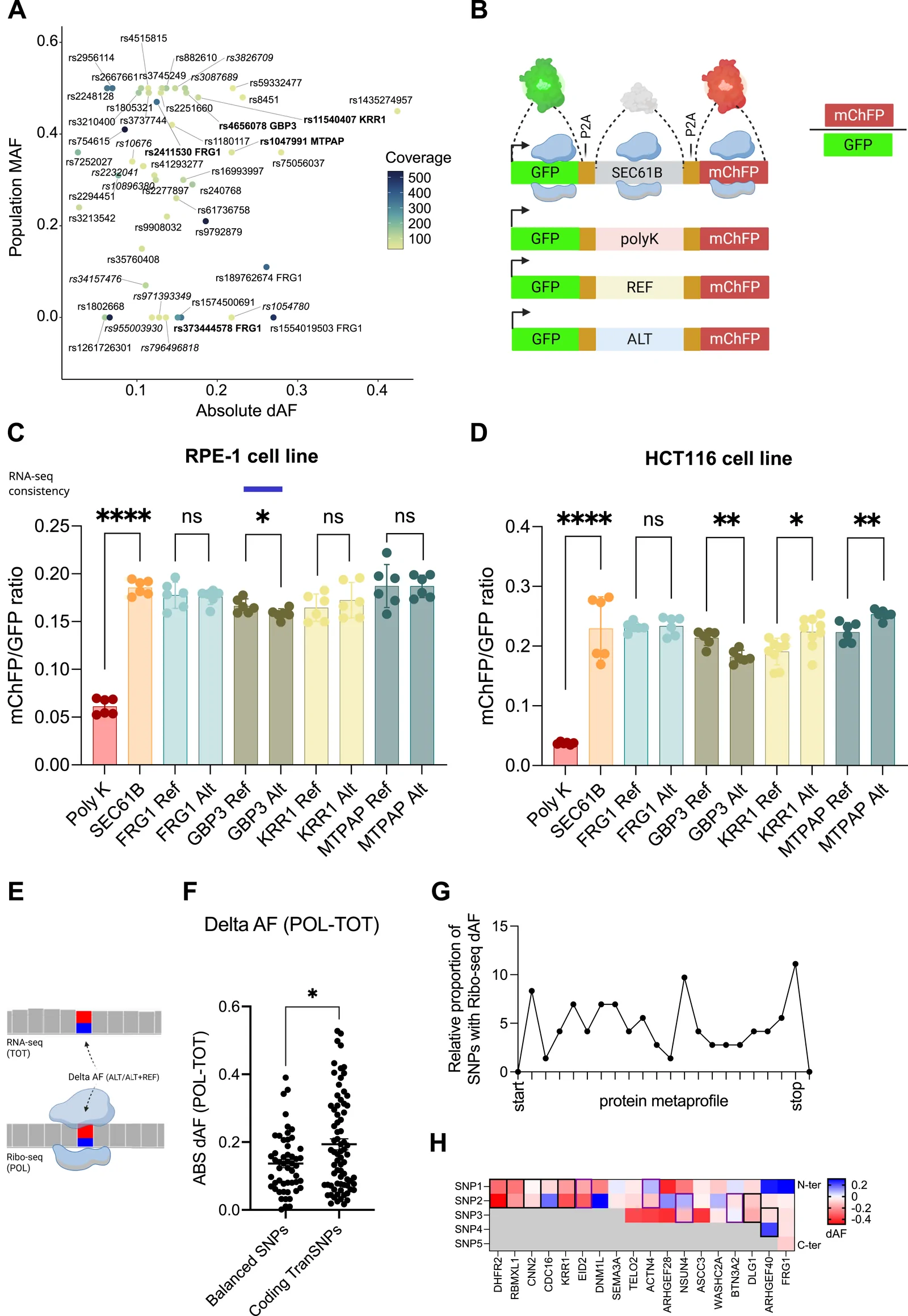
Coding RPE-1 tranSNPs influence translation in a dual-fluorescence reporter assay. **(A)** Bubble plot representing the correlation between absolute dAF, global population minor allelic frequency, and coverage to guide the prioritization of coding RPE-1 tranSNPs. The highest absolute dAF value across conditions was plotted for each tranSNP. The selected tranSNPs for the reporter assay are shown in bold; the tranSNPs excluded from further analyses are shown in italics. **(B)** Schematic representation of the ribosome stalling construct. The cloned sequences of interest (ALT/REF) were flanked by P2A sites to allow ribosome skipping and the cleavage of the three peptides separately from a single mRNA. The *SEC61B* sequence in the ribosome stalling construct was used as a negative control. The ratio of mCherry to GFP signal intensities provides a measure of ribosome-scanning efficiency along the sequence of interest. **(C)** mChFP/GFP ratios for the four selected tranSNPs measured in RPE-1 cells. Consistency with RNA-seq data is highlighted by a blue segment. **(D)** mChFP/GFP ratios for the four selected tranSNPs measured in HCT116 cells. The fluorescence values were obtained using Operetta High Content System. The blue bar indicates the consistency between relative allele activities and dAF measured in RNA-seq data. Data represent the mean ± SD of at least two independent experiments. ns, not significant, *P <0.05, **P <0.01, ****P <0.0001, unpaired *t* test. **(E)** Scheme of the approach used to assess publicly available Ribo-seq data to quantify allelic imbalance on ribosome footprints. An IGV snapshot is used to visualize how the dAF is calculated. The matched RNA-seq data are used as total (TOT) RNA coverage, whereas the Ribo-seq data are used as polysomal RNA. **(F)** Comparison of dAF for the control and tranSNP groups. Ns = not significant; *P <0.05, ordinary one-way ANOVA with Tukey’s multiple comparisons test. **(G)** Metaprofile of the distribution of 73 coding tranSNPs in 41 proteins from RPE-1 datasets (PRJNA281177 and PRJNA528026) (Table S2). Ticks in the x-axis correspond to 5% increments in relative protein length. **(H)** dAF calculated from RNA-seq and Ribo-seq data for the 18 proteins for which haplotype data consisting of at least two SNPs were retrievable. The data are ordered on the y-axis by haplotype length, whereas on the x-axis, SNPs are positioned relative to their positions in the primary sequence. Haplotypes for which phasing data could be retrieved from our RNA-seq data are highlighted in thicker black boxes when the reference and alternative alleles are in cis, and in purple when in trans.

We also selected four RPE-1 candidate tranSNPs located in the CDS under the hypothesis that missense or silent changes in the CDS could influence translation elongation, ribosome pausing, or even stalling (Tsai et al, 2014). Similar to the approach with UTR SNPs, candidates were selected based on the magnitude of AF imbalance, allele frequency in the population, RNA-seq coverage, host gene function, and, when available, concordance with other tranSNPs within the same mRNA, informed by haplotype analysis (Fig 4A and Table S3).

Table S3. Information on coding, missense, and synonymous TranSNPs.

For the four chosen candidates, located in the *FRG1*, *KRR1*, *GBP3*, and *MTPAP* genes, we cloned 109-nucleotide-long fragments of CDS, coding for 33 amino acids and containing either the reference or the alternative SNP allele into a dual-fluorescence reporter plasmid. Specifically, the sequences of interest were cloned between a GFP and an mCherry and flanked by two P2A sequences (Kriachkov et al, 2023) to produce a single long open reading frame but enabling the separate production of GFP and mCherry polypeptides (Fig 4B). An alteration in the efficiency of decoding by the ribosome at the level of a cloned intervening sequence is expected to reduce the mCherry signal, which lies downstream, and, thus, the overall mChFP/GFP ratio. The *SEC61B* sequence, which is known to allow efficient readthrough, was used as a negative control for stalling. A strong positive control for stalling, containing a sequence coding for a stretch of 17 consecutive lysine residues (polyK), was also used. The mChFP/GFP ratios for all cloned sequences were comparable to those of the negative control, as expected; however, slight allele-specific differences, particularly in HCT116, were apparent (Fig 4C and D). Notably, we found consistency with RNA-seq data in RPE-1 cells for the tranSNP in *GBP3*, as the alternative allele was predicted to be associated with higher translation, thus less ribosome pausing or stalling (Fig 4C).

**Figure S6.**
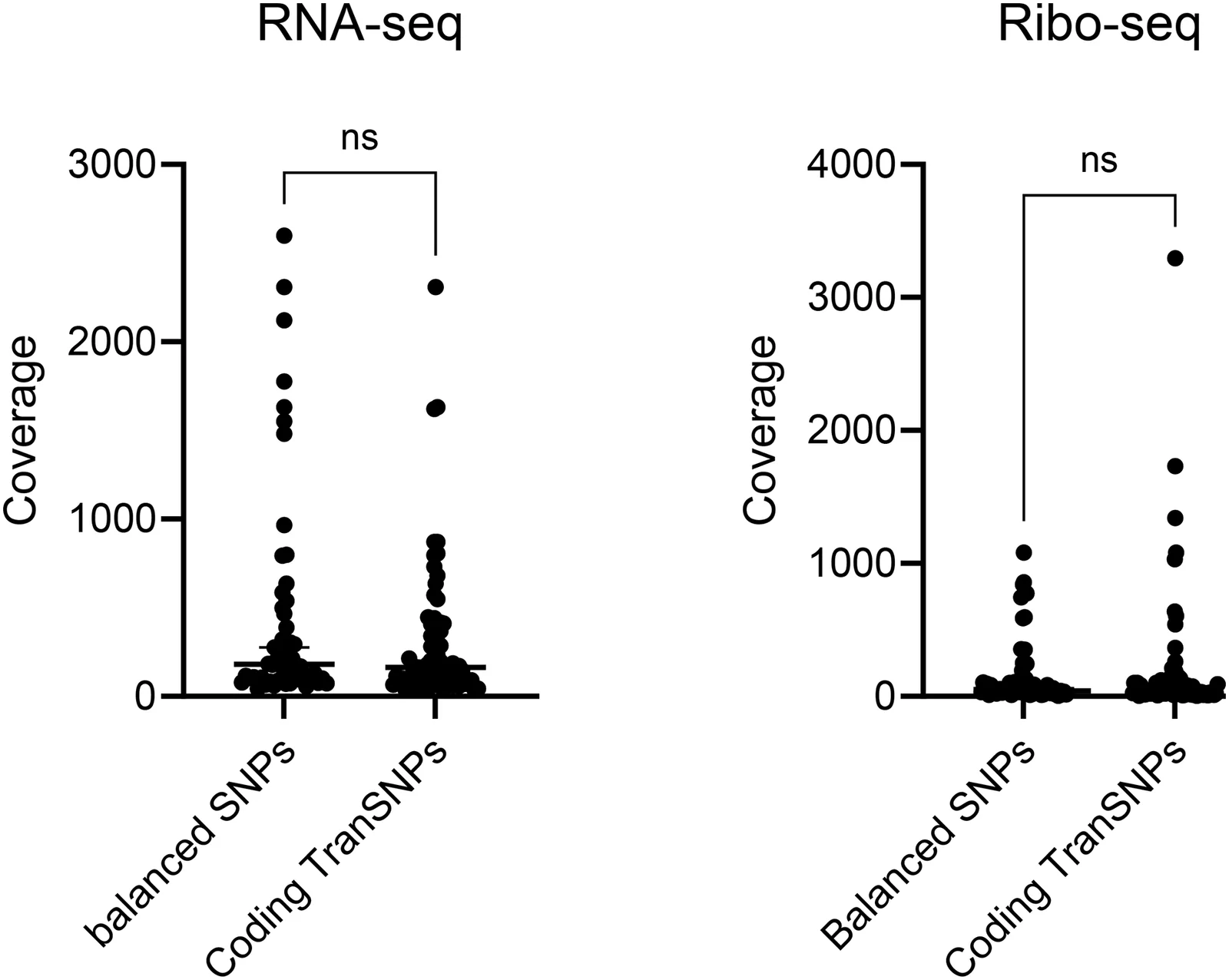
Extended dAF data for 80S-associated haplotypes (related to Fig 2). RNA-seq and Ribo-seq coverage (related to Fig 4). RNA-seq and Ribo-seq coverage for a control group of heterozygous RPE-1 SNPs not showing allelic imbalance, and the tranSNPs grouped into silent and missense alleles.

We also analyzed two publicly available RNA-seq coupled with Ribo-seq data in RPE-1 (BioProjects PRJNA281177 and PRJNA528026) (Tanenbaum et al, 2015; Haneke et al, 2020) and used them as proxies for total RNA and polysomal RNA, respectively, to independently validate the allelic imbalances observed with our method (Fig 4E). Data were obtained for 73 tranSNPs (Table S3). Results were compared with a control group of 52 RPE-1 SNPs showing balanced AF in our datasets (Table S3). A significantly higher dAF was observed for the tranSNP group (Fig 4F), whereas no differences in the number of RNA-seq or Ribo-seq reads were noted (Fig S6). We also asked whether allelic imbalance in Ribo-seq data is associated with mRNA topography. Overall, there was no clear positional bias for the tranSNPs that could be analyzed, except for a slight enrichment toward the C-ter (Fig 4G). Next, we looked more specifically at instances of tranSNP-containing haplotypes. Ribo-seq data were available for 18 cases. Haplotype phasing data could be retrieved for 11 of the 18 SNP pairs, of which seven were in cis (Table S2). We hypothesized that if a single SNP within a haplotype causes translation pausing or stalling, the direction of dAF may be complementary between preceding and following SNPs. For three of them (*CDC16*, *EID2*, and *ARHGEF40*), the haplotype dAF data support this conjecture (Fig 4H). There was also a trend for increasing dAF values along the primary transcript.

### The effect of missense tranSNPs can be explored by allele-specific proteomics

**Figure 5.**
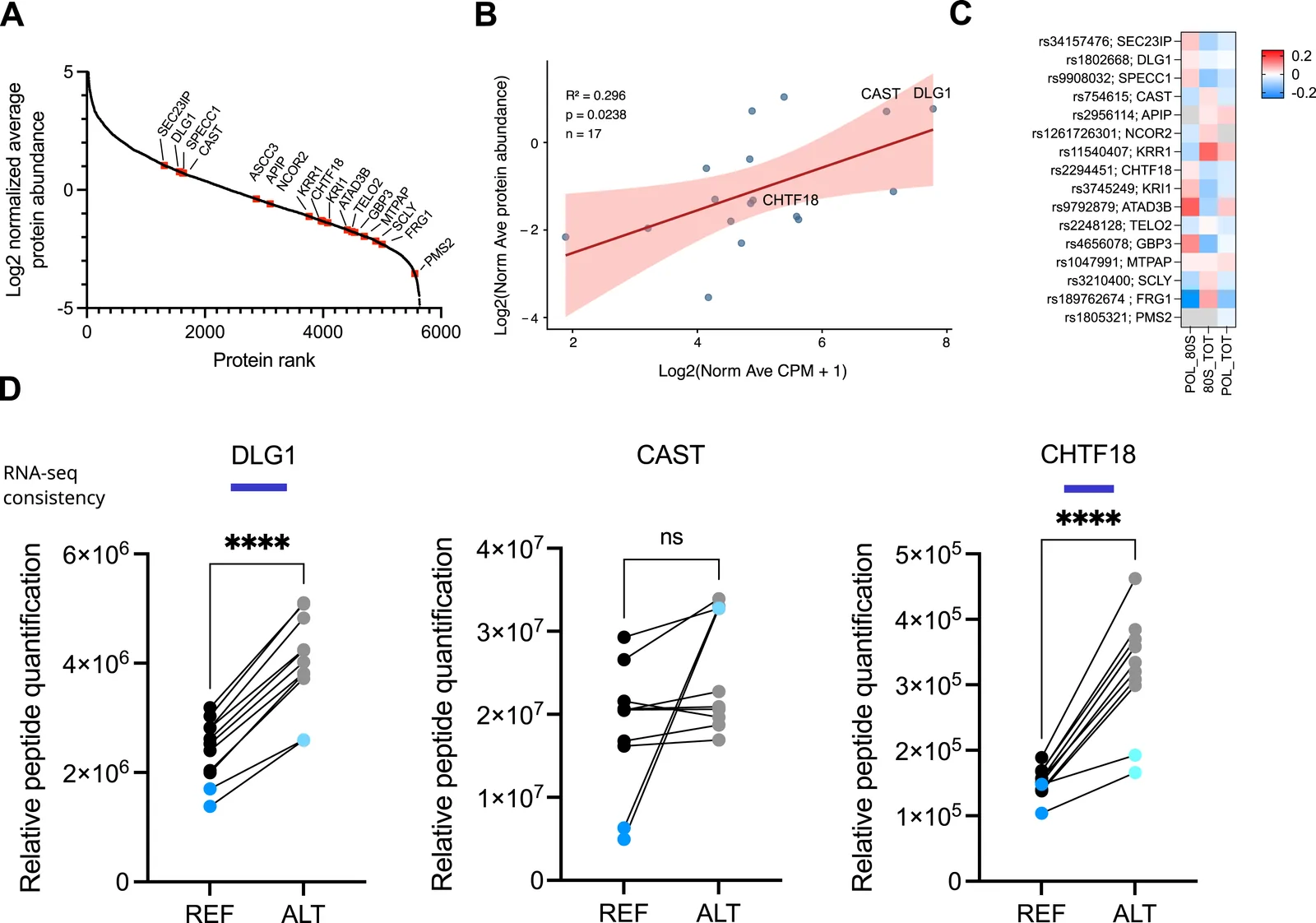
Exploration of missense tranSNPs by label-free proteomics. **(A)** Whole proteome using label-free shotgun analysis. Relative protein abundance was ranked based on the aggregated results of three biological replicates, each analyzed by three injections. The ranked positions of proteins from the list of missense tranSNPs are highlighted in red. **(B)** Comparison between mRNA expression (CPM, average of the biological replicates) and relative protein abundance from RPE-1 whole proteome analysis. Although 17 proteins with missense tranSNPs were identified, in only three cases, highlighted by the protein name, peptides comprising the biallelic variant were detected by mass spectrometry. **(C)** Heatmap showing allelic fraction imbalance data (dAF) for the coding tranSNP candidates across POL-TOT, 80S-TOT, and POL-80S comparisons. **(D)** Relative quantification of the reference (REF) and alternative (ALT) peptides for *DLG1*, *CAST*, and *CHTF18* by label-free targeted analysis, based on three biological replicates. The two data points in blue refer to analyses of technical replicates of a whole protein lysate sample, whereas those in black refer to three biological samples prepared by in-gel digestion, each tested with two technical replicates. ns, not significant; ****P < 0.0001, two-tailed paired *t* test. The consistency of AF imbalance measured via RNA-seq with the relative peptide quantification is indicated by a blue segment underneath the gene name.

For coding tranSNPs that cause missense changes, it is, in principle, possible to explore allele-specific differences at endogenous levels by leveraging allele-specific proteomics. We recently validated this approach using the EIF4H gene and the rs1554710467 SNV as an example (Hamadou et al, 2026). In the RPE-1 dataset, 40 missense tranSNPs in 35 proteins were found (Table S3). Compared with standard protein identification by mass spectrometry, a specific peptide containing the SNP allele has to be detected. Furthermore, an added complexity arises from potential differences in the ionization efficiency of the two peptides corresponding to the tranSNP alleles, and, in the case of amino acid changes affecting arginine residues, also allele-specific differences in peptide length upon trypsin digestion. Indeed, of the 40 missense changes, 12 involved arginine residues in either the reference or the alternative alleles (Table S3). However, LysC, GluC, and chymotrypsin were less efficient than trypsin. Using label-free shotgun proteomics, peptides from 17 of the 35 proteins were identified (Fig 5A). However, only in four cases peptides spanning the biallelic position were detected (Fig 5B), and in the case of FRG1, only the reference peptide was found. In general, transcripts and protein relative abundance were correlated, but abundance alone did not predict the likelihood of finding the desired peptide for allelic quantification (Fig 5B). Allelic imbalance data for the candidate coding tranSNPs (Fig 5C) also showed that the tranSNP-containing genes for which both allelic peptides could be detected by mass spectrometry (*DLG1*, *CAST*, and *CHTF18*) did not necessarily exhibit the highest magnitude of allelic imbalance. Besides using a whole protein lysate, to enrich for the *DLG1*, *CAST*, and *CHTF18* proteins, total extracts were resolved by SDS–PAGE, and stained with Coomassie, and gel bands were excised, cut, destained, and in-gel–digested using trypsin (see Materials and Methods). A targeted approach was then chosen to obtain relative quantifications of the reference and alternative peptides (Fig 5D). We did not detect allele-specific differences for *CAST*, and there was large variation between whole-lysate and in-gel digestion data. Instead, we found significant differences in the relative quantification of the two alleles for *DLG1* and *CHTF18*. Notably, the direction of the imbalance was consistent with the RNA-seq data in both cases (Fig 5D).

### TranSNPs are associated with allele-dependent RBP interactions and clinical outcomes

To begin exploring trans-factors potentially involved in allele-specific interactions by targeting tranSNP-containing sequences, binding predictions were generated using TESS software (Schug, 2008), starting with a list of 53 RBPs with a consensus motif (Cook et al, 2011). These were used to compute binding scores for the reference and alternative alleles for 280 tranSNPs (Table S1). RBP motifs overlapping a SNP site were found for 194 of the 280 cases (Table S4a). This list was further refined by filtering for a binding score of at least 75% of the maximum score for one of the two alleles and for at least 50% change in the binding score for one of the two alleles. This conservative approach led to a shortlist of 110 among the 280 tranSNPs with at least one prediction of allele-prevalent binding by RBPs. A total of 137 allele-prevalent binding predictions were obtained (Table S4b) for 11 RBPs. About 65% of the predicted sites mapped to coding exons. Focusing on the 17 tranSNPs for which validation reporter assays were developed, predictions of allele-specific binding were obtained for seven SNP sites and five different RBPs (Table 1). The SNP location, a summary of the allele exhibiting higher translation potential inferred from the RNA-seq, and the allele preferentially bound based on the score comparisons are included. Although highly exploratory, the consistency between SRSF1 allele preference and translation potential aligns with the literature (Maslon et al, 2014). The rs3737577 prediction in *S1PR1A* could also be consistent with an inhibitory effect of ELAV1/HuR when binding to the 5′ UTR, in contrast to its typical stabilizing effect at 3′ UTR binding sites (Meng et al, 2005).

Table S4. RNA-binding protein motifs analysis.

**Table 1.** Predictions of allele-specific or allele-prevalent binding for tranSNPs validated in the reporter assays.

rsID	Gene	Location	RBP	REF allele RBP score	ALT allele RBP score	Max score	% of change	Allele with higher translation (RNA-seq)	Allele preferentially bound (RBP scores)
rs140546414	ABL1	3′ UTR	MBNL1	4.38	None	6.87	−63.73	ALT	REF
rs140546414	ABL1	3′ UTR	RBMX	0.13	5.17	5.86	86.02	ALT	ALT
rs12983784	ELAVL1	3′ UTR	SFRS1	1.87	4.19	4.62	50.26	ALT	ALT
rs12983784	ELAVL1	3′ UTR	EIF4B	3.25	8.98	10.49	54.61	ALT	ALT
rs1357255885	PTPN14	3′ UTR	EIF4B	2.22	8.88	9.98	66.73	REF	ALT
rs1357255885	PTPN14	3′ UTR	RBMX	5.86	0.82	5.86	−86.02	REF	REF
rs1063181	SPRYD7	5′ UTR	SFRS1	3.57	1.25	4.62	−50.26	REF	REF
rs1063181	SPRYD7	5′ UTR	EIF4B	5.75	None	7.08	−81.14	REF	REF
rs1404595755	WASHC1	3′ UTR	FUS	0.60	7.89	7.89	92.44	REF	ALT
rs1404595755	WASHC1	3′ UTR	SFRS1	3.57	1.25	4.62	−50.26	REF	REF
rs3737577	S1PR1	5′ UTR	ELAVL1	2.08	4.40	4.40	52.73	REF	ALT
rs4656078	GBP3	CDS	RBMX	4.91	0.52	5.86	−74.90	ALT	REF

**Figure 6.**
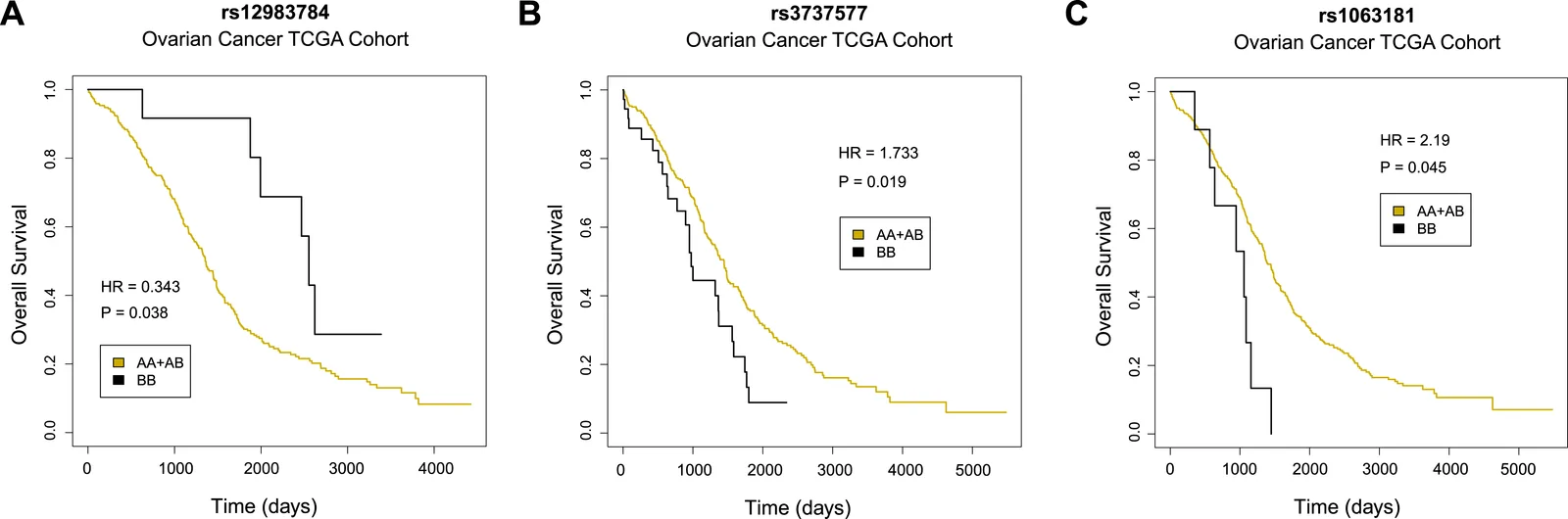
TranSNPs can be correlated with distinct clinical outcomes in TCGA. The rs12983784 (A), rs3737577 (B), and rs1063181 (C) genotypes stratify ovarian cancer patients across a TCGA cohort for the overall survival endpoint. Kaplan–Meier curves plotting the overall survival under a recessive model (B = ALT allele) are presented along with the hazard ratio (HR) and the *P*-values.

Finally, we interrogated TCGA data (Liu et al, 2018) using germline genetic information to explore the potential clinical relevance of the identified tranSNPs. Among the seven tranSNPs validated by reporter assays and for which allele-specific RBP binding was predicted, four were associated with clinical outcomes under either a recessive or a dominant model for the alternative allele across overall survival, disease-free survival, disease-free interval, and progression-free interval endpoints (Table S5). Three of them, rs12983784 in the *ELAVL1* 3′ UTR, rs3737577 in the *S1PR1* 5′ UTR, and rs1063181 in the *SPRYD7* 5′ UTR, had prognostic value in ovarian cancer (Fig 6A–C), suggesting a potential functional link between RNA–protein interactions and clinical phenotypes.

Table S5. Correlation of tranSNP status and TCGA clinical endpoints.

## Discussion

Our study establishes that allele-associated differences in translational mRNA regulation can also be identified and studied in nontransformed cells. The dataset generated in RPE-1 cells revealed that monosome-enriched fractions facilitated the identification of tranSNPs (Figs 1, 2, and S1). This result is not entirely unexpected, as the TOT we previously used as the sole comparison with the polysomal RNAs is a reconstituted pool of all cytoplasmic fractions separated by sucrose gradient ultracentrifugation (Figs 1A and S1D); hence, it also contains polysomes. In principle, focusing on sucrose gradient fractions free of polysomes and monosomes would facilitate the identification of alleles affecting translation potential. However, polysome profiling coupled to qRT-PCR typically shows that light fractions contain a low proportion of the mRNA signal, as confirmed for RPE-1 (Fig S4), which may negatively affect RNA-seq quality and SNP calling.

The translational potential of the 80S monosome remains debated (Heyer & Moore, 2016; Schneider et al, 2022), and recent evidence in *Drosophila melanogaster* indicates that it can be highly tissue-dependent (Blandy et al, 2025). This feature would limit the possibility of generalizing the use of 80S-bound mRNAs as a proxy for untranslated mRNAs. Interestingly, in *Drosophila*, 80S monosomes appeared to be translationally active during developmental stages or in organs where translation regulation requires more precise control, involving structured 5′ UTRs or upstream ORFs. Conversely, 80S monosomes appeared inactive in the testis and ovary, where polysomes were the major sites of protein synthesis (Blandy et al, 2025). Oncogenic signaling, or, conversely, nutrient starvation or hypoxia, can impact both global and mRNA-specific translation (Koritzinsky et al, 2006; Mossmann et al, 2018; Lee et al, 2020). Hence, cell context and specific conditions or perturbations may alter the translational potential of the 80S and its value for pursuing the genetic bases of allelic differences in translational regulation.

Moreover, although we observed that the comparison of POL-80S provides higher resolution in tranSNP identification in RPE-1 cells, we further found that comparing 80S with total cytoplasmic mRNAs has higher discriminatory potential, consisting in larger dAF, than comparing polysomal mRNAs with TOT (Fig 1H), suggesting that the polysomal presence in the TOT fraction contributes more than the 80S in the total cytoplasmic mRNA fraction in this cell line.

We used luciferase-based assays to evaluate allele-specific differences in translation efficiency for 5′ and 3′ UTR targets. Differences in the magnitude of the effect of the cloned fragments were observed between 5′ UTR and 3′ UTR constructs across both RPE-1 and HCT116 cells. These can be attributable to the use of two different plasmid scaffolds that contain a distinct constitutive promoter and may differ in the translation efficiency of the reporter: pGL3p plasmid was used for cloning the 5′ UTR targets, and pGL4.13, harboring a codon-optimized Firefly luciferase, for cloning the 3′ UTR targets. Both types of plasmids express high levels of the reporter, which could reduce the assay’s dynamic range; however, we adopted double normalization of the light units, using both the control Renilla luciferase activity and the relative levels of firefly mRNA measured by qRT-PCR in the same extracts used for the luminescence assay. Importantly, in RPE-1 cells tranSNPs selected exploiting the 80S monosome sequencing were validated more efficiently. As noted, comparing results in RPE-1 and HCT116 showed examples of striking cell line differences in allelic impact, suggesting strong context-dependent effects possibly related to the different use of the 80S monosome or the relative expression of trans-factors.

Tissue-specific differences in the expression of RBPs and microRNAs, tRNAs, epitranscriptomic writers, and readers can underlie differences in the kinetics or extent of pre-mRNA splicing, mRNA export, subcellular localization, polyadenylation, translation initiation, and elongation, all features that can also impact the potential to identify distinct flavors of biallelic mRNA fate (Fabbri et al, 2021; Xiang et al, 2024; Koletsou & Huppertz, 2025; Luo et al, 2025; Tian et al, 2026). These posttranscriptional regulatory layers may lead to tranSNP positional biases along mRNA sequences in cases of heterozygous haplotypes, as we sought to explore in this study. High-depth RNA-seq, paired-end protocols, and especially long-read sequencing may enable the identification of tranSNPs at the transcript rather than the gene level in future studies.

Although the analysis pipeline was specifically developed to prioritize functional SNPs in posttranscriptional gene regulation, establishing the causal effects of cis-alleles remains challenging because of low throughput and limitations of reporter assays that must account for posttranscriptional mRNA regulation. In this respect, fluorescence-based assays are, in principle, superior to luminescence-based assays, although relative sensitivity can be a limitation. Even more complex is the identification of causative trans-factors. For example, the validation of allele-specific binding of an RBP to a tranSNP-containing site requires either experiments with allelic resolution in cells, such as a RIP protocol followed by bound RNA analysis, or edited isogenic cells homozygous for the two tranSNP genotypes, or else the comparison of binding in a collection of heterologous cells that already harbor distinct tranSNP genotypes, but may also differ for the relative expression of target RBPs. In this respect, the data we presented on predicted binding sites of tranSNP-altered RBPs represent an exploratory first step toward a mechanistic understanding. Indeed, we do not rule out the possibility that additional mechanisms, distinct from affected RBP binding, may be important in regulating allele-specific translation; tranSNPs may alter upstream ORFs by altering start or stop codons within Kozak sequence motifs, alter m6A or polyadenylation sites, impact microRNA binding, or affect RNA secondary structures. Our investigation on the prognostic features in TCGA cancer patient cohorts stratified based on the tranSNP genotype is also very preliminary. These limitations notwithstanding, the fact that among the 17 candidate tranSNPs chosen for validation, allele-specific differences in reporter activity, RBP binding, and patientsʼ survival were found for two of them suggests that our dataset can be a useful resource for discovering functional genetic variants, particularly in the UTRs of mRNAs.

The translation efficiency of coding tranSNPs has been assessed by a dual-fluorescence assay originally designed to detect instances of ribosome stalling (Kriachkov et al, 2023); however, because we could not control in detail the readout of the assay, this term was used to broadly indicate events of ribosome pausing or stalling, which may eventually affect translation elongation. We observed allele-specific differences in ribosome stalling capacity. The magnitude of the effect was expectedly modest compared with the positive control, which features a strong stalling sequence consisting of 17 consecutive lysine residues. We estimated that the magnitude of the effect of the single amino acid substitutions we studied is similar to the impact observed with 10 copies of a GR-coding hexanucleotide repeat derived from c9orf72 intron 1 (Kriachkov et al, 2023). We also measured allelic imbalance of a number of tranSNPs using publicly available Ribo-seq and RNA-seq datasets. However, it has to be taken into consideration that Ribo-seq footprints do not distinguish monosomes from polysomes, although the positional resolution, coupled to haplotype phasing when multiple heterozygous SNPs are present, could help exploring whether allele-specific effects are related to differences in translation initiation, or in ribosome pausing at specific sequence contexts, or to reduced availability of one allelic transcript to the translation machinery caused, for example, by mRNA compartmentalization. In this latter case, dAF consistent in direction along the mRNA sequence are expected, whereas in the former two cases, a change in the direction of the dAF along the mRNA sequence could be anticipated. In fact, if a tranSNP represents a ribosome pause site, ribosomes might queue before it, but not after (Fig 4H). Although limited by sequencing depth and resulting coverage at SNP sites, our results suggest that Ribo-seq data, coupled with biallelic markers in the CDS, could reveal genetic variants affecting subtle mRNA translation features. It would be interesting to explore whether variations to the Ribo-seq protocol, such as polysome sequencing (Yoshikawa et al, 2018) or RiboLace (Clamer et al, 2018), that is, methods that enable the isolation of translating ribosomes, could increase the allelic resolution at tranSNP sites. The isolation of specific polysome species using optimized sucrose gradients (Liang et al, 2018) represents further improvements to the experimental pipeline. In addition to 80S monosome sequencing, the fractions lighter than ribosomal subunits and the 40S fraction could be used as a source of cytoplasmic mRNA not undergoing translation. However, considering the typical distribution of relative mRNA abundance along the sucrose gradient profile (Panda et al, 2017; Hamadou et al, 2025), mRNA species in those fractions may be underrepresented. Finally, the isolation and sequencing of nuclear polyadenylated mRNAs could also be considered alongside subpolysomal, 80S, and polysomal cytoplasmic fractions to collectively capture distinct aspects of posttranscriptional and translational mRNA regulation, empowered by the biallelic marker resolution.

tranSNPs that result in missense changes are potentially amenable to validation at the endogenous level in heterozygous cells using allele-specific proteomics. We recently validated this approach using a missense SNV in EIF4H (Hamadou et al, 2026). Here, we explored whether a simplified mass spectrometry approach that did not include isotype-labeled synthetic peptide spike-ins for each chosen target could still enable tranSNP validation. Results confirmed that MS sensitivity remains suboptimal, especially given the need to quantify a specific, biallelic peptide and the complexity introduced by missense changes affecting arginine or methionine residues, which can alter peptide properties, such as ionization or oxidation. In fact, starting from 35 candidate proteins, only in three cases could both the reference and alternative peptide relative quantifications be obtained, and a significant imbalance was observed in two of them (Fig 5). For the *CAST* target, the difference in the relative quantification of the reference peptide between whole lysates and SDS–PAGE and in-gel digestion remains unexplained. We also acknowledge that conclusions about mechanisms inferred solely from our proteomics data are limited because we did not measure protein half-life, which is another possible mechanism that could account for differences in protein levels resulting from missense tranSNPs.

Nutlin treatment in RPE-1 cells led to broad changes in the transcriptome consistent with p53 activation and cell cycle arrest (Andrysik et al, 2017; Rizzotto et al, 2020; Tiu et al, 2021). The treatment-dependent changes in gene expression were largely coordinated at the transcriptional and translational levels, although a trend toward high-magnitude repression was apparent in the translatome (Fig S2). Nutlin treatment was shown to alter global translation control by modulating p53-dependent EIF4E-BP1 and ribosome biogenesis factors, RBPs, and microRNAs (Hermeking, 2012; Marcel et al, 2013, 2018; Tiu et al, 2021; Lindström et al, 2022). However, nutlin treatment of RPE-1 cells did not result in a global change in the detection of tranSNPs. Hence, our analysis pipeline can be applied in a broader context, beyond cancer research, as a tool to map genetically driven interindividual sources of phenotypic diversity and provide clues to guide mechanistic studies.

## Materials and Methods

### Cell culture

The human noncancer retinal pigmented epithelial RPE-1 cells were cultured in Dulbecco’s modified Eagle’s medium and Ham’s F12 supplemented with 10% FBS, 100 U/ml penicillin, 100 mg/ml streptomycin antibiotic mix (GIBCO), and 1% L-glutamine (GIBCO). The cells were maintained in a humidified atmosphere at 37°C and 5% CO_2_. Cells were split twice a week.

### RNA extraction

RNA was extracted using NucleoSpin RNA Extraction Kit (Macherey-Nagel) according to the manufacturer’s instructions. RNA purity and concentration were assessed by NanoDrop spectrophotometer (Thermo Fisher Scientific).

### cDNA and qRT-PCR

RNA was retrotranscribed using RevertAid First Strand cDNA Synthesis Kit (Thermo Fisher Scientific) and random hexamer primers according to the manufacturer’s instructions. One μg of RNA was retrotranscribed, and the cDNA was diluted 1:8 in water, equivalent to a final concentration of 6.25 ng/μl. qRT-PCR was performed using the PowerUp SYBR mix (Applied Biosystems) in 10 μl, using 12.5 ng of cDNA as input and 0.4 μM of each primer. Primers were designed using Primer-BLAST (NCBI) and are listed in Table S6. The QuantStudio 5 instrument was used to amplify the cDNA and measure gene expression. Technical triplicates were performed, and average Cq values were normalized to housekeeping genes (dCt) and to controls (ddCt) when possible.

Table S6. Table of the primers used in this study.

### Polysome profiling and RNA-seq

Cells were grown in 150-mm dishes to 70–80% confluence and incubated for 10 min with 100 μg/ml cycloheximide at 37°C to block protein synthesis and immobilize ribosomes on mRNAs. Cells were then rinsed with ice-cold PBS 1X containing 50 μg/ml (or 100 μg/ml) cycloheximide, scraped, and pelleted. The cell pellet was lysed with 600 μl of ice-cold lysis buffer (20 mM Tris–HCl, pH 7.5, 130 mM KCl, 30 mM MgCl_2_, 2.5 mM DTT, 0.2% NP-40, 0.5% sodium deoxycholate, 0.2 mg/ml heparin, 0.2 U/μl RNasin Ribonuclease Inhibitor (Promega), cOmplete Mini Protease Inhibitor Cocktail 1X (Roche), 100 μg/ml cycloheximide) by incubation on ice for 10 min and centrifugation at 12,000*g* at 4°C for 10 min to remove cellular debris and nuclei. The cytoplasmic lysate was then loaded on a 15–50% linear sucrose gradient dissolved in salt buffer (100 mM NaCl, 20 mM Tris–HCl, pH 7.5, and 5 mM MgCl_2_) and processed by ultracentrifugation in a Beckman SW41 Ti swinging bucket rotor at 40,000 rpm for 1 h and 40 min at 4°C. The gradient was then fractionated in 1 ml fractions, and the absorbance was measured at 254 nm by the Teledyne ISCO model 160 gradient analyzer equipped with a UA-6 UV/VIS detector. RNA was extracted from the fractions using TRIzol based on the manufacturer’s instructions. RNA-seq libraries were prepared from 1,000 ng of total RNA using the Illumina Stranded mRNA library preparation kit, according to the manufacturer’s instructions. Libraries were sequenced at the CIBIO NGS Core Facility on an Illumina NovaSeq 6000 system using an S1 flow cell in paired-end mode, generating approximately 100 million 2 × 100 bp read pairs per sample. To validate allelic imbalances identified by RNA-seq, RNA isolated from selected polysomal profile fractions was reverse-transcribed, as described in the previous section, and regions encompassing selected tranSNPs were PCR-amplified using GoTaq Green Master Mix (Promega) and specific primers (Table S6). The resulting amplicons were purified with QIAquick PCR & Gel Clean-Up Kit and subsequently analyzed by Sanger sequencing using Mix2Seq Kit (Eurofins Genomics) according to the manufacturer’s instructions. Electropherograms were analyzed using Fiji software. AF were calculated by measuring the area under the peaks corresponding to the tranSNP nucleotides. Values were normalized by accounting for the relative incorporation efficiency of each nucleotide during sequencing, based on the local sequence context.

### RNA-seq data processing, differential expression analysis, and tranSNP identification

Paired-end RNA-seq data from total, 80S/monosomal, and polysome-associated RNA fractions were processed from raw FASTQ files to generate BAM files for gene-level expression analysis. Adapter sequences and low-quality bases were removed using Trimmomatic (Bolger et al, 2014). Trimmed reads were aligned to the human reference genome GRCh38 using STAR (Dobin et al, 2013), generating one BAM file for each sample.

Gene-level expression was quantified by counting reads mapping to genes annotated in the reference genome using the Rsubread R package (Liao et al, 2019). Differential gene expression analysis was performed with the edgeR R package (Robinson et al, 2010) to identify genes showing significant expression changes across RNA fractions. Pairwise comparisons were performed among total, 80S/monosomal, and polysome-associated RNA fractions. Genes were considered differentially expressed based on statistical significance, defined as adjusted *P* <0.01, effect size, defined as absolute log2 fold change >1, and average expression signal, defined as logCPM >0. Functional enrichment analysis was performed separately on the lists of up-regulated and down-regulated genes obtained from each pairwise comparison using the Metascape web portal (Zhou et al, 2019), which integrates Gene Ontology terms, pathway databases, and additional functional annotations to identify significantly overrepresented biological processes and molecular pathways. For tranSNP identification, the aligned BAM files were subjected to additional post-alignment processing to improve the reliability of allele-specific read counts at SNP positions. PCR duplicates were removed using the Picard MarkDuplicates module. The resulting BAM files were then processed with GATK to perform RNA-seq–specific refinement steps, including splitting of reads spanning splice junctions, local realignment, and base-quality recalibration. These additional processing steps were applied specifically for AF estimation and were not used for differential gene expression analysis.

TranSNP identification was performed following the workflow previously developed for the detection of single-nucleotide variants associated with allele-specific translation potential (Valentini et al, 2021) and implemented as described recently (Hamadou et al, 2025). Briefly, postprocessed BAM files were interrogated at dbSNP-annotated SNP positions located in exonic and UTRs. Pileup at SNP positions was performed using ASEQ (Romanel et al, 2015) with parameters mode = 0, mbq = 20, and mrq = 20. AF were calculated as the number of reads supporting the alternative allele divided by the total number of reads supporting the alternative and reference alleles. A 20–80% AF range to identify heterozygous SNPs was adopted from the validated default parameters of the ASEQ pileup-based method used for genotyping known variants (Romanel et al, 2015). Importantly, we have successfully applied the same criterion in several previous studies in which accurate genotyping of common SNPs was critical (Prandi et al, 2014; Carrot-Zhang et al, 2020; Orlando et al, 2022; Scandino et al, 2025), further supporting its robustness across different experimental settings. These empirical cutoffs provide a conservative classification of heterozygous sites, allowing variants with AF between 0.2 and 0.8 to be retained even in the presence of moderate reference-mapping bias or allelic imbalance, whereas variants with more extreme AF are more consistent with homozygous calls or potentially unreliable genotype assignments. Heterozygous SNPs with sufficient read coverage were retained, and AF were compared across total, 80S/monosomal, and polysome-associated RNA fractions. Candidate tranSNPs were defined as variants showing consistent allelic imbalance between RNA fractions across biological replicates.

### Cloning of tranSNP-containing sequences for reporter assays

Forward and reverse primers containing the restriction sites and the sequence of interest (insert) were designed (sequences are reported in Table S6), resuspended to a final concentration of 100 μM, and simultaneously annealed and phosphorylated by mixing 1:1 (8.5 μl each) in a final volume of 20 μl with 2 μl of 10× DNA ligase buffer (Thermo Fisher Scientific) and 0.5 μl of PNK kinase (New England Biolabs). Annealed inserts were diluted 1:500 in water. In the meantime, backbone plasmids (about 1 μg) were double-digested using restriction enzymes for 3 h at 37°C using 0.5 μl of each enzyme in a final volume of 20 μl. The digestion product was purified from gel using Promega Gel Extraction and PCR Clean-Up Kit and quantified using the NanoDrop Spectrophotometer (Thermo Fisher Scientific). About 50 ng of digested backbone was incubated with 1–2 μl of annealed insert (or water in the insert negative sample) and 2 μl of DNA T4 Ligase (Thermo Fisher Scientific) in a final volume of 20 μl for 2–3 h at 22°C. The ligation product was entirely transformed into competent DH5α *Escherichia coli* bacteria (see the Bacterial transformation section), and all bacteria were plated onto ampicillin Liquid Broth Agar plates and incubated overnight at 37°C. Single colonies were picked and cultured in 5 ml of Liquid Broth supplemented with ampicillin overnight at 37°C in a shaking incubator. Plasmid DNA was extracted using PureYield Plasmid Miniprep System (Promega) and quantified, and the correct insertion was verified via diagnostic digestion and Sanger sequencing.

### Luciferase-based reporter assays

5,000 cells were seeded in a 96-well plate format in 100 μl of medium. After 24 h, cells were transfected with 75 ng of DNA from the cloned constructs on Firefly backbone plasmids (pGL3 promoter for 5′ UTR targets and linker pGL4.13 for 3′ UTR targets; see Table S6; a “linker” was added to the pGL4 plasmid to generate an EcoRI and an NdeI restriction site around the XbaI site), and 25 ng of DNA from the Renilla luciferase plasmid. Plasmid DNA was diluted in Opti-MEM Reduced Serum Medium (Thermo Fisher Scientific), and FuGENE HD (Promega) was used as a transfection reagent in a 4:1 ratio. The transfection reaction was assembled in a 5 μl of volume and incubated at room temperature for 15 min before being added dropwise to the cells. At 48 h post-transfection, cells were washed with 150 μl of 1X PBS and lysed using 50 μl per well of passive lysis buffer 1X with shaking at 500 rpm for 15 min (Orbi-Shaker, Benchmark Scientific). Passive lysis buffer and the following reagents were part of Dual-Luciferase Reporter Assay System. 10 μl of lysate was then transferred into white 384-well plates. 10 μl of Luciferase Assay Reagent, which is the Firefly luciferase substrate, was added to each well. Upon brief shaking, the Firefly luciferase signal was detected using Varioskan LUX Multimode Microplate Reader (Thermo Fisher Scientific). Three readings were performed. STOP&Glo reagent was then diluted 1:50 in the provided buffer, and 10 μl was added to each well. After brief shaking, the Renilla luciferase signal was detected using the same instrument; three more readings were performed. Data were analyzed by calculating the ratio of Firefly to Renilla luminescence signals for each well. The remaining 40 μl of lysate was collected, and RNA was extracted using TRIzol (Invitrogen). RNA was then quantified and retrotranscribed, and Firefly mRNA was amplified via qRT-PCR (as in the cDNA and qRT-PCR section).

### Ribosome stalling assay

109-nucleotide-long sequences (coding for 33 amino acids) comprising either the alternative or the reference version of the CDS tranSNPs were cloned in dual-reporter ribosome stalling plasmid constructs (Kriachkov et al, 2023) upstream of an mCherry and downstream of a GFP sequence, separated by P2A sequences which induce the ribosomes to skip the formation of peptide bonds without interrupting translation, allowing the translation of separate peptides from a single mRNA (Lin et al, 2013). Cells were seeded in a 96-well plate format and, after 24 h, transfected with 100 ng of the above-mentioned ribosome stalling plasmids using the FuGENE transfection reagent (Promega) in a 4:1 ratio. A known staller sequence (coding for 17 consecutive lysines, polyK) cloned in the same plasmid was used as a positive control. A *SEC61B* sequence (106 amino acids), which is known to allow readthrough, was cloned into the same plasmid and used as a negative control. GFP and mCherry signals, and digital phase contrast were detected at 24, 36, and 48 h post-transfection using Operetta High Content Imaging System (PerkinElmer). GFP and mCherry intensities were used to calculate mChFP/GFP ratios, which were used as proxies for ribosome pausing, stalling, or, more generally, alterations in translation elongation.

### Proteomic analysis: sample preparation and selection of peptides for targeted proteomics (PRM)

RPE-1 cells were harvested and processed in RIPA buffer for 30 min at 4°C, followed by centrifugation to remove cellular debris. Protein concentration was quantified via the BCA assay. For each sample, 20 μg of protein underwent reduction with 10 mM Tris(2-carboxyethyl)phosphine for 30 min at room temperature using 20 mM iodoacetamide, at 25°C for 30 min in the dark. Protein digestion was conducted using carboxylate-modified magnetic beads (GE Healthcare) according to the SP3 protocol (Hughes et al, 2019). Briefly, washed beads were mixed with proteins at a 1:10 (wt/wt) ratio. Acetonitrile (ACN) was added to a final concentration of 70% (Vol/Vol), and the mixture was incubated at room temperature for 18 min. After magnetic separation and washing (twice with 70% ethanol, once with ACN), beads were resuspended in digestion buffer (50 mM NH_4_HCO_3_, 5 mM CaCl_2_) containing different proteases (LysC, GluC, chymotrypsin, or trypsin) at a 1:15 enzyme-to-protein ratio. After an overnight incubation at 37°C, peptides were desalted using BioSPE PurePep Tips (Affinisep), according to the manufacturer’s instructions. To enrich proteins of interest, lysates were separated via SDS–PAGE using Bolt 10% Bis-Tris minigels (Thermo Fisher Scientific) and visualized with Imperial Protein Stain (Thermo Fisher Scientific). Gel regions corresponding to 25–45 kD and 70–100 kD were excised and minced into ∼1 mm^3^ fragments. These pieces were destained using a 1:1 mixture of 100 mM NH_4_HCO_3_ and ACN, followed by dehydration in 100% ACN and centrifugal evaporation (SpeedVac). Proteins were reduced with 10 mM DTT and alkylated with 55 mM iodoacetamide. After washes with 100 NH_4_HCO_3_ and ACN, the dried gel pieces were saturated with trypsin (12.5 ng/μl) on ice for 45 min. Proteolysis was conducted overnight at 37°C. Peptides were recovered through sequential extraction using 30% ACN/3% trifluoroacetic acid and pure ACN. The pooled extracts were concentrated via SpeedVac, acidified to pH < 2.5 with 1% trifluoroacetic acid, and desalted using laboratory-made C18 StageTips. Peptide samples were reconstituted in 0.1% formic acid and stored at −20°C before shotgun and PRM analysis.

Selected proteins were subjected to in silico tryptic digestion to identify peptide sequences harboring the SNPs of interest. Because peptide selection was constrained by the specific biallelic variant sites, it was not always feasible to meet all standard criteria typically applied for the choice of optimal quantotypic peptides for PRM assays (Rauniyar, 2015). In particular, the only sequence-related limitation was the unavoidable inclusion of cysteine residues requiring carbamidomethylation. For each candidate peptide, the most suitable precursor charge state was determined through manual evaluation of mass spectrometric performance, considering factors such as signal-to-noise ratio, transition intensity, chromatographic peak shape, and integrated peak area.

### Proteomic analysis: LC-MS/MS and data analysis

For shotgun label-free quantification, digested peptide samples were separated using a Dionex 3000 nanoLC RSLC system (Thermo Fisher Scientific) coupled to a heated reversed-phase analytical column (75 μm internal diameter, 1.7 μm particle size, 100 Å pore size; CoAnn Technologies) maintained at 40°C. Chromatographic separation was performed using a binary mobile phase system composed of 0.1% formic acid in water (buffer A) and 0.1% formic acid in ACN (buffer B). Peptides were eluted at a flow rate of 300 nl/min using the following gradient: 5–25% buffer B over 80 min, 25–40% over 3 min, 40–98% over 2 min, followed by a 15-min wash at 98% buffer B. Eluting peptides were analyzed on an Orbitrap Fusion Tribrid mass spectrometer (Thermo Fisher Scientific) operating in data-dependent acquisition mode, with a spray voltage of 2.1 kV and an ion transfer tube temperature of 200°C. Full MS scans were acquired in the Orbitrap at a resolution of 120,000 (FWHM at m/z 200), with an AGC target of 4 × 10^6^ and a maximum injection time of 246 ms. Survey scans covered a mass range of m/z 250–1,500. Dynamic exclusion was enabled with a 40-s exclusion window. Each survey scan was followed by MS/MS fragmentation using higher energy collisional dissociation at 30% normalized collision energy within a 3-s duty cycle. MS/MS scans were acquired either in the Orbitrap or in the ion trap, with maximum injection times of 54 ms and 35 ms, respectively, and an AGC target of 2.5 × 10^4^.

For targeted PRM analysis, peptides containing the specific biallelic amino acid variants were monitored. Liquid chromatography conditions were identical to those used for shotgun analyses. Selected precursor ions were included in an isolation list containing peptide masses and charge states (Table S7). PRM scans were acquired in the Orbitrap at a resolution of 120,000, with an AGC target of 4.0 × 10^5^, a maximum injection time of 246 ms, and higher energy collisional dissociation fragmentation at 30% normalized collision energy. To minimize sample carryover and ensure consistent analytical performance, blank injections and quality control samples were analyzed between runs. Instrument performance was continuously monitored throughout the study using QCloud (Chiva et al, 2018).

Table S7. Ion isolation list for PRM analysis.

For data analysis, extracted ion chromatograms corresponding to all monitored transitions were manually inspected using Xcalibur Qual Browser (Thermo Fisher Scientific). For shotgun proteomic datasets, peptide identification was performed using Proteome Discoverer (version 3.1) with the reviewed human proteome database from UniProt (Proteome ID: UP000005640; downloaded in October 2025), supplemented with custom variant-containing protein sequences and a common contaminant database. Database searches were carried out using the MASCOT search engine (server version 2.6.2.0), applying a precursor mass tolerance of 10 ppm and fragment ion tolerances of 0.02 or 0.6 D, depending on the acquisition settings. Proteolytic specificity was defined according to the enzyme used (trypsin, GluC, or LysC), allowing up to two missed cleavages. Carbamidomethylation of cysteine residues was set as a fixed modification, whereas methionine oxidation and protein N-terminal acetylation were included as variable modifications. Peptide and protein identifications were filtered using a false discovery rate of <1% at the peptide–spectrum match, peptide, and protein levels. Known contaminants were removed from downstream analyses. Protein abundance values were log_2_-transformed, and normalization was performed based on the mean sample abundance to reduce technical variability associated with differences in sample loading (Aguilan et al, 2020).

For PRM experiments, peptide peak areas were quantified using the open-source software Skyline (daily version 26.1) (MacLean et al, 2010). Transitions were selected based on fragment ion intensity, interference assessment, and reproducibility across replicates. Only transitions consistently detected across replicates were retained, whereas inconsistent or interfered transitions were manually excluded. Four fragment ions per peptide were ultimately used for quantification. PRM data were normalized using total ion current, and the ratios between alternative (ALT) and reference (REF) peptide peak areas were calculated and log-transformed before statistical analysis. The complete shotgun proteomics and PRM datasets were deposited in the ProteomeXchange Consortium through the PRIDE partner repository (Yasset et al, 2025).

## Supplementary Material

Reviewer comments

## Data Availability

The RNA-seq data generated in this study have been deposited in the NCBI Sequence Read Archive under BioProject accession number PRJNA1456805. The shotgun proteomics data were deposited in the PRIDE repository (ProteomeXchange accession: PXD078075). PRM data were deposited in PANORAMA (PXD077902).

## References

[bib1] Aguilan JT, Kulej K, Sidoli S (2020) Guide for protein fold change and p-value calculation for non-experts in proteomics. Mol Omics 16: 573–582. 10.1039/d0mo00087f 32968743

[bib2] Andrysik Z, Galbraith MD, Guarnieri AL, Zaccara S, Sullivan KD, Pandey A, MacBeth M, Inga A, Espinosa JM (2017) Identification of a core TP53 transcriptional program with highly distributed tumor suppressive activity. Genome Res 27: 1645–1657. 10.1101/gr.220533.117 PMC5630028 28904012

[bib3] Biever A, Glock C, Tushev G, Ciirdaeva E, Dalmay T, Langer JD, Schuman EM (2020) Monosomes actively translate synaptic mRNAs in neuronal processes. Science 367: eaay4991. 10.1126/science.aay4991 32001627

[bib4] Biffo S, Ruggero D, Santoro MM (2024) The crosstalk between metabolism and translation. Cell Metab 36: 1945–1962. 10.1016/j.cmet.2024.07.022 PMC11586076 39232280

[bib5] Blandy A, Hopes T, Vasconcelos EJR, Turner A, Fatkhullin B, Agapiou M, Fontana J, Aspden JL (2025) Translational activity of 80S monosomes varies dramatically across different tissues. Nucleic Acids Res 53: gkaf292. 10.1093/nar/gkaf292 PMC12056609 40331628

[bib6] Bolger AM, Lohse M, Usadel B (2014) Trimmomatic: A flexible trimmer for Illumina sequence data. Bioinformatics 30: 2114–2120. 10.1093/bioinformatics/btu170 PMC4103590 24695404

[bib7] Carrot-Zhang J, Chambwe N, Damrauer JS, Knijnenburg TA, Robertson AG, Yau C, Zhou W, Berger AC, Huang K-L, Newberg JY, (2020) Comprehensive analysis of genetic ancestry and its molecular correlates in cancer. Cancer Cell 37: 639–654.e6. 10.1016/j.ccell.2020.04.012 PMC7328015 32396860

[bib8] Cenik C, Cenik ES, Byeon GW, Grubert F, Candille SI, Spacek D, Alsallakh B, Tilgner H, Araya CL, Tang H, (2015) Integrative analysis of RNA, translation, and protein levels reveals distinct regulatory variation across humans. Genome Res 25: 1610–1621. 10.1101/gr.193342.115 PMC4617958 26297486

[bib9] Chin LJ, Ratner E, Leng S, Zhai R, Nallur S, Babar I, Muller RU, Straka E, Su L, Burki EA, (2008) A SNP in a let-7 microRNA complementary site in the KRAS 3′ untranslated region increases non-small cell lung cancer risk. Cancer Res 68: 8535–8540. 10.1158/0008-5472.CAN-08-2129 PMC2672193 18922928

[bib10] Chiva C, Olivella R, Borràs E, Espadas G, Pastor O, Solé A, Sabidó E (2018) QCloud: A cloud-based quality control system for mass spectrometry-based proteomics laboratories. PLoS One 13: e0189209. 10.1371/journal.pone.0189209 PMC5764250 29324744

[bib11] Clamer M, Tebaldi T, Lauria F, Bernabò P, Gómez-Biagi RF, Marchioretto M, Kandala DT, Minati L, Perenthaler E, Gubert D, (2018) Active ribosome profiling with RiboLace. Cell Rep 25: 1097–1108.e5. 10.1016/j.celrep.2018.09.084 30355487

[bib12] Cook KB, Kazan H, Zuberi K, Morris Q, Hughes TR (2011) RBPDB: A database of RNA-binding specificities. Nucleic Acids Res 39: D301–D308. 10.1093/nar/gkq1069 PMC3013675 21036867

[bib13] Dobin A, Davis CA, Schlesinger F, Drenkow J, Zaleski C, Jha S, Batut P, Chaisson M, Gingeras TR (2013) STAR: Ultrafast universal RNA-seq aligner. Bioinformatics 29: 15–21. 10.1093/bioinformatics/bts635 PMC3530905 23104886

[bib14] Fabbri L, Chakraborty A, Robert C, Vagner S (2021) The plasticity of mRNA translation during cancer progression and therapy resistance. Nat Rev Cancer 21: 558–577. 10.1038/s41568-021-00380-y 34341537

[bib15] Fernández-Calero T, Davyt M, Perelmuter K, Chalar C, Bampi G, Persson H, Tosar JP, Hafstað V, Naya H, Rovira C, (2020) Fine-tuning the metabolic rewiring and adaptation of translational machinery during an epithelial-mesenchymal transition in breast cancer cells. Cancer Metab 8: 8. 10.1186/s40170-020-00216-7 PMC7368990 32699630

[bib16] Hamadou MH, Alunno L, Venturelli T, Valentini S, Dalfovo D, Lorenzini F, Mattivi A, Vigorito V, Grupelli GP, *Matte A, et al* (2025) A common germline 3’UTR variant controls TDDIT4 translation efficiency and stress responses in cancer cells. *iScience* In press. 10.1016/j.isci.2026.117707

[bib17] Hamadou M, Alunno L, Peroni D, Pancher M, Mazza F, Venturelli T, Belli R, Dassi E, Romanel A, Inga A (2026) Polysomal profiling coupled to allele-specific proteomics reveals an EIF4H TranSNP allele possessing higher mRNA translation potential. Mol Cell Proteomics 25: 101535. 10.1016/j.mcpro.2026.101535 PMC13010432 41722779

[bib18] Haneke K, Schott J, Lindner D, Hollensen AK, Damgaard CK, Mongis C, Knop M, Palm W, Ruggieri A, Stoecklin G (2020) CDK1 couples proliferation with protein synthesis. J Cell Biol 219: e201906147. 10.1083/jcb.201906147 PMC7054999 32040547

[bib19] Hermeking H (2012) MicroRNAs in the p53 network: Micromanagement of tumour suppression. Nat Rev Cancer 12: 613–626. 10.1038/nrc3318 22898542

[bib20] Heyer EE, Moore MJ (2016) Redefining the translational status of 80S monosomes. Cell 164: 757–769. 10.1016/j.cell.2016.01.003 26871635

[bib21] Huang E, Fu T, Zhang L, Yan G, Yamamoto R, Terrazas S, Nguyen TL, Gonzalez-Figueroa C, Khanbabaei A, Bahn JH, (2025) Genetic variants affecting RNA stability influence complex traits and disease risk. Nat Genet 57: 2578–2588. 10.1038/s41588-025-02326-8 40913182

[bib22] Hughes CS, Moggridge S, Müller T, Sorensen PH, Morin GB, Krijgsveld J (2019) Single-pot, solid-phase-enhanced sample preparation for proteomics experiments. Nat Protoc 14: 68–85. 10.1038/s41596-018-0082-x 30464214

[bib23] Jia L, Mao Y, Ji Q, Dersh D, Yewdell JW, Qian SB (2020) Decoding mRNA translatability and stability from the 5′ UTR. Nat Struct Mol Biol 27: 814–821. 10.1038/s41594-020-0465-x 32719458

[bib24] Jia JJ, Lahr RM, Solgaard MT, Moraes BJ, Pointet R, Yang AD, Celucci G, Graber TE, Hoang HD, Niklaus MR, (2021) MTORC1 promotes TOP mRNA translation through site-specific phosphorylation of LARP1. Nucleic Acids Res 49: 3461–3489. 10.1093/nar/gkaa1239 PMC8034618 33398329

[bib25] Khan D, Sharathchandra A, Ponnuswamy A, Grover R, Das S (2013) Effect of a natural mutation in the 5′ untranslated region on the translational control of p53 mRNA. Oncogene 32: 4148–4159. 10.1038/onc.2012.422 23027126

[bib26] Koletsou E, Huppertz I (2025) RNA-binding proteins as versatile metabolic regulators. NPJ Metab Heal Dis 3: 1. 10.1038/s44324-024-00044-z PMC12091694 40603707

[bib27] Koritzinsky M, Magagnin MG, van den Beucken T, Seigneuric R, Savelkouls K, Dostie J, Pyronnet S, Kaufman RJ, Weppler SA, Voncken JW, (2006) Gene expression during acute and prolonged hypoxia is regulated by distinct mechanisms of translational control. EMBO J 25: 1114–1125. 10.1038/sj.emboj.7600998 PMC1409715 16467844

[bib28] Kriachkov V, Ormsby AR, Kusnadi EP, McWilliam HEG, Mintern JD, Amarasinghe SL, Ritchie ME, Furic L, Hatters DM (2023) Arginine-rich C9ORF72 ALS proteins stall ribosomes in a manner distinct from a canonical ribosome-associated quality control substrate. J Biol Chem 299: 102774. 10.1016/j.jbc.2022.102774 PMC9830226 36481270

[bib29] Lee P, Chandel NS, Simon MC (2020) Cellular adaptation to hypoxia through hypoxia inducible factors and beyond. Nat Rev Mol Cell Biol 21: 268–283. 10.1038/s41580-020-0227-y PMC7222024 32144406

[bib30] Li Q, Makri A, Lu Y, Marchand L, Grabs R, Rousseau M, Ounissi-Benkalha H, Pelletier J, Robert F, Harmsen E, (2013) Genome-wide search for exonic variants affecting translational efficiency. Nat Commun 4: 2260. 10.1038/ncomms3260 PMC3749366 23900168

[bib31] Li Z, Chen L (2023) Predicting functional consequences of SNPs on mRNA translation via machine learning. Nucleic Acids Res 51: 7868–7881. 10.1093/nar/gkad576 PMC10450169 37427781

[bib32] Liang S, Bellato HM, Lorent J, Lupinacci FCS, Oertlin C, van Hoef V, Andrade VP, Roffé M, Masvidal L, Hajj GNM, (2018) Polysome-profiling in small tissue samples. Nucleic Acids Res 46: E3. 10.1093/nar/gkx940 PMC5758873 29069469

[bib33] Liao Y, Smyth GK, Shi W (2019) The R package Rsubread is easier, faster, cheaper and better for alignment and quantification of RNA sequencing reads. Nucleic Acids Res 47: e47. 10.1093/nar/gkz114 PMC6486549 30783653

[bib34] Lin YJ, Huang LH, Huang CT (2013) Enhancement of heterologous gene expression in flammulina velutipes using polycistronic vectors containing a viral 2A cleavage sequence. PLoS One 8: e59099. 10.1371/journal.pone.0059099 PMC3597617 23516605

[bib35] Lindström MS, Bartek J, Maya-Mendoza A (2022) p53 at the crossroad of DNA replication and ribosome biogenesis stress pathways. Cell Death Differ 29: 972–982. 10.1038/s41418-022-00999-w PMC9090812 35444234

[bib36] Liu J, Lichtenberg T, Hoadley KA, Poisson LM, Lazar AJ, Cherniack AD, Kovatich AJ, Benz CC, Levine DA, Lee AV, (2018) An integrated TCGA pan-cancer clinical data resource to drive high-quality survival outcome analytics. Cell 173: 400–416.e11. 10.1016/j.cell.2018.02.052 PMC6066282 29625055

[bib38] Luo H, Kharas MG, Jaffrey SR (2025) N6-Methyladenosine: An RNA modification as a central regulator of cancer. Nat Rev Cancer 26: 118–136. 10.1038/s41568-025-00889-6 PMC13142248 41360987

[bib39] MacLean B, Tomazela DM, Shulman N, Chambers M, Finney GL, Frewen B, Kern R, Tabb DL, Liebler DC, MacCoss MJ (2010) Skyline: An open source document editor for creating and analyzing targeted proteomics experiments. Bioinformatics 26: 966–968. 10.1093/bioinformatics/btq054 PMC2844992 20147306

[bib40] Marcel V, Ghayad SE, Belin S, Therizols G, Morel AP, Solano-Gonzàlez E, Vendrell JA, Hacot S, Mertani HC, Albaret MA, (2013) P53 acts as a safeguard of translational control by regulating fibrillarin and rRNA methylation in cancer. Cancer Cell 24: 318–330. 10.1016/j.ccr.2013.08.013 PMC7106277 24029231

[bib41] Marcel V, Van Long FN, Diaz JJ (2018) 40 Years of research put P53 in translation. Cancers (Basel) 10: 152. 10.3390/cancers10050152 PMC5977125 29883412

[bib42] Maslon MM, Heras SR, Bellora N, Eyras E, Cáceres JF (2014) The translational landscape of the splicing factor SRSF1 and its role in mitosis. Elife 3: e02028. 10.7554/eLife.02028 PMC4027812 24842991

[bib43] Mata J, Marguerat S, Bähler J (2005) Post-transcriptional control of gene expression: A genome-wide perspective. Trends Biochem Sci 30: 506–514. 10.1016/j.tibs.2005.07.005 16054366

[bib44] Meng Z, King PH, Nabors BL, Jackson NL, Chen CY, Emanuel PD, Blume SW (2005) The ELAV RNA-stability factor HuR binds the 5′-untranslated region of the human IGF-IR transcript and differentially represses cap-dependent and IRES-mediated translation. Nucleic Acids Res 33: 2962–2979. 10.1093/nar/gki603 PMC1140080 15914670

[bib45] Mitschka S, Mayr C (2022) Context-specific regulation and function of mRNA alternative polyadenylation. Nat Rev Mol Cell Biol 23: 779–796. 10.1038/s41580-022-00507-5 PMC9261900 35798852

[bib46] Mossmann D, Park S, Hall MN (2018) mTOR signalling and cellular metabolism are mutual determinants in cancer. Nat Rev Cancer 18: 744–757. 10.1038/s41568-018-0074-8 30425336

[bib47] Orellana EA, Siegal E, Gregory RI (2022) tRNA dysregulation and disease. Nat Rev Genet 23: 651–664. 10.1038/s41576-022-00501-9 PMC11170316 35681060

[bib48] Orlando F, Romanel A, Trujillo B, Sigouros M, Wetterskog D, Quaini O, Leone G, Xiang JZ, Wingate A, Tagawa S, (2022) Allele-informed copy number evaluation of plasma DNA samples from metastatic prostate cancer patients: The PCF-select consortium assay. NAR Cancer 4: zcac016. 10.1093/narcan/zcac016 PMC9154344 35664542

[bib49] Panda A, Martindale J, Gorospe M (2017) Polysome fractionation to analyze mRNA distribution profiles. Bio Protoc 7: e2126. 10.21769/BioProtoc.2126 PMC5431591 28516123

[bib50] Prandi D, Baca SC, Romanel A, Barbieri CE, Mosquera JM, Fontugne J, Beltran H, Sboner A, Garraway LA, Rubin MA, (2014) Unraveling the clonal hierarchy of somatic genomic aberrations. Genome Biol 15: 439. 10.1186/s13059-014-0439-6 PMC4167267 25160065

[bib51] Rahaman S, Schiffelholz N, Mittal N, Fröhlich KE, Zavolan M, Becskei A (2025) Heat shock induces silent ribosomes and reorganizes mRNA turnover. Cell Rep 44: 116447. 10.1016/j.celrep.2025.116447 41108687

[bib52] Rauniyar N (2015) Parallel reaction monitoring: A targeted experiment performed using high resolution and high mass accuracy mass spectrometry. Int J Mol Sci 16: 28566–28581. 10.3390/ijms161226120 PMC4691067 26633379

[bib53] Rizzotto D, Zaccara S, Rossi A, Galbraith MD, Andrysik Z, Pandey A, Sullivan KD, Quattrone A, Espinosa JM, Dassi E, (2020) Nutlin-induced apoptosis is specified by a translation program regulated by PCBP2 and DHX30. Cell Rep 30: 4355–4369.e6. 10.1016/j.celrep.2020.03.011 PMC7182397 32234473

[bib54] Robert F, Pelletier J (2018) Exploring the impact of single-nucleotide polymorphisms on translation. Front Genet 9: 507. 10.3389/fgene.2018.00507 PMC6218417 30425729

[bib55] Robichaud N, Sonenberg N, Ruggero D, Schneider RJ (2019) Translational control in cancer. Cold Spring Harb Perspect Biol 11: a032896. 10.1101/cshperspect.a032896 PMC6601465 29959193

[bib81] Robinson MD, McCarthy DJ, Smyth GK (2010) edgeR: A Bioconductor package for differential expression analysis of digital gene expression data. Bioinformatics 26: 139–140. 10.1093/bioinformatics/btp616 PMC2796818 19910308

[bib56] Romanel A, Lago S, Prandi D, Sboner A, Demichelis F (2015) ASEQ: Fast allele-specific studies from next-generation sequencing data. BMC Med Genomics 8: 9. 10.1186/s12920-015-0084-2 PMC4363342 25889339

[bib57] Ruggero D (2013) Translational control in cancer etiology. Cold Spring Harb Perspect Biol 5: a012336. 10.1101/cshperspect.a012336 PMC3552512 22767671

[bib58] Sample PJ, Wang B, Reid DW, Presnyak V, McFadyen IJ, Morris DR, Seelig G (2019) Human 5′ UTR design and variant effect prediction from a massively parallel translation assay. Nat Biotechnol 37: 803–809. 10.1038/s41587-019-0164-5 PMC7100133 31267113

[bib59] Scandino R, Nardone A, Casiraghi N, Galardi F, Genovese M, Romagnoli D, Paoli M, Biagioni C, Tonina A, Migliaccio I, (2025) Enabling sensitive and precise detection of ctDNA through somatic copy number aberrations in breast cancer. NPJ Breast Cancer 11: 25. 10.1038/s41523-025-00739-6 PMC11890748 40057527

[bib60] Schneider C, Erhard F, Binotti B, Buchberger A, Vogel J, Fischer U (2022) An unusual mode of baseline translation adjusts cellular protein synthesis capacity to metabolic needs. Cell Rep 41: 111467. 10.1016/j.celrep.2022.111467 36223745

[bib61] Schug J (2008) Using TESS to predict transcription factor binding sites in DNA sequence. Curr Protoc Bioinformatics 21: 2.6.1–2.6.15. 10.1002/0471250953.bi0206s21 18428685

[bib62] Shetty S, Hofstetter J, Battaglioni S, Ritz D, Hall MN (2023) TORC1 phosphorylates and inhibits the ribosome preservation factor Stm1 to activate dormant ribosomes. EMBO J 42: e112344. 10.15252/embj.2022112344 PMC9975950 36691768

[bib64] Tanenbaum ME, Stern-Ginossar N, Weissman JS, Vale RD (2015) Regulation of mRNA translation during mitosis. Elife 4: e07957. 10.7554/eLife.07957 PMC4548207 26305499

[bib65] Thoreen CC, Chantranupong L, Keys HR, Wang T, Gray NS, Sabatini DM (2012) A unifying model for mTORC1-mediated regulation of mRNA translation. Nature 485: 109–113. 10.1038/nature11083 PMC3347774 22552098

[bib66] Tian B, Yu S, Zhang Q (2026) Regulation of gene expression by alternative polyadenylation in health and disease. Nat Rev Genet 27: 385–404. 10.1038/s41576-025-00928-w PMC13037395 41540274

[bib67] Tiu GC, Kerr CH, Forester CM, Krishnarao PS, Rosenblatt HD, Raj N, Lantz TC, Zhulyn O, Bowen ME, Shokat L, (2021) A p53-dependent translational program directs tissue-selective phenotypes in a model of ribosomopathies. Dev Cell 56: 2089–2102.e11. 10.1016/j.devcel.2021.06.013 PMC8319123 34242585

[bib68] Tsai A, Kornberg G, Johansson M, Chen J, Puglisi JD (2014) The dynamics of SecM-induced translational stalling. Cell Rep 7: 1521–1533. 10.1016/j.celrep.2014.04.033 PMC4059775 24836001

[bib69] Valentini S, Marchioretti C, Bisio A, Rossi A, Zaccara S, Romanel A, Inga A (2021) TranSNPs: A class of functional SNPs affecting mRNA translation potential revealed by fraction-based allelic imbalance. iScience 24: 103531. 10.1016/j.isci.2021.103531 PMC8666669 34917903

[bib70] Volpe E, Colantoni A, Corda L, Di Tommaso E, Pelliccia F, Ottalevi R, Guarracino A, Licastro D, Faino L, Capulli M, (2025) The reference genome of the human diploid cell line RPE-1. Nat Commun 16: 7751. 10.1038/s41467-025-62428-z PMC12432231 40940351

[bib71] Wang G, van der Walt JM, Mayhew G, Li YJ, Züchner S, Scott WK, Martin ER, Vance JM (2008) Variation in the miRNA-433 binding site of FGF20 confers risk for Parkinson disease by overexpression of α-synuclein. Am J Hum Genet 82: 283–289. 10.1016/j.ajhg.2007.09.021 PMC2427225 18252210

[bib72] Warner JR, Knopf PM, Rich A (1963) A multiple ribosomal structure in protein synthesis. Proc Natl Acad Sci U S A 49: 122–129. 10.1073/pnas.49.1.122 PMC300639 13998950

[bib73] Wu Q, Bazzini AA (2023) Translation and mRNA stability control. Annu Rev Biochem 92: 227–245. 10.1146/annurev-biochem-052621-091808 37001134

[bib74] Xiang JS, Schafer DM, Rothamel KL, Yeo GW (2024) Decoding protein–RNA interactions using CLIP-based methodologies. Nat Rev Genet 25: 879–895. 10.1038/s41576-024-00749-3 PMC12356153 38982239

[bib75] Xiong Y, Wang A, Kang Y, Shen C, Hsieh CY, Hou T (2025) mRNABERT: Advancing mRNA sequence design with a universal language model and comprehensive dataset. Nat Commun 16: 10371. 10.1038/s41467-025-65340-8 PMC12644827 41285802

[bib76] Yasset P-R, Bandla C, Kundu DJ, Kamatchinathan S, Bai J, Hewapathirana S, John NS, Prakash A, Walzer M, Wang S, (2025) The PRIDE database at 20 years: 2025 Update. Nucleic Acids Res 53: D543–D553. 10.1093/nar/gkae1011 PMC11701690 39494541

[bib77] Yoshikawa H, Larance M, Harney DJ, Sundaramoorthy R, Ly T, Owen-Hughes T, Lamond AI (2018) Efficient analysis of mammalian polysomes in cells and tissues using ribo mega-SEC. Elife 7: e36530. 10.7554/eLife.36530 PMC6086667 30095066

[bib78] Zhao BS, Roundtree IA, He C (2016) Post-transcriptional gene regulation by mRNA modifications. Nat Rev Mol Cell Biol 18: 31–42. 10.1038/nrm.2016.132 PMC5167638 27808276

[bib79] Zheng D, Persyn L, Wang J, Liu Y, Ulloa-Montoya F, Cenik C, Agarwal V (2025) Predicting the translation efficiency of messenger RNA in mammalian cells. Nat Biotechnol 44: 783–796. 10.1038/s41587-025-02712-x PMC12323635 40715456

[bib80] Zhou Y, Zhou B, Pache L, Chang M, Khodabakhshi AH, Tanaseichuk O, Benner C, Chanda SK (2019) Metascape provides a biologist-oriented resource for the analysis of systems-level datasets. Nat Commun 10: 1523. 10.1038/s41467-019-09234-6 PMC6447622 30944313

